# Transcriptomic Coordination in the Human Metabolic Network Reveals Links between n-3 Fat Intake, Adipose Tissue Gene Expression and Metabolic Health

**DOI:** 10.1371/journal.pcbi.1002223

**Published:** 2011-11-03

**Authors:** Melissa J. Morine, Audrey C. Tierney, Ben van Ommen, Hannelore Daniel, Sinead Toomey, Ingrid M. F. Gjelstad, Isobel C. Gormley, Pablo Pérez-Martinez, Christian A. Drevon, Jose López-Miranda, Helen M. Roche

**Affiliations:** 1 Nutrigenomics Research Group, UCD Conway Institute, University College Dublin, Dublin, Ireland; 2 TNO Quality of Life, Zeist, The Netherlands; 3 Molecular Nutrition Unit, Center of Life and Food Science, Technical University of Munich, Freising-Weihenstephan, Germany; 4 Department of Nutrition, Institute of Basic Medical Sciences, Faculty of Medicine, University of Oslo, Oslo, Norway; 5 Department of Endocrinology, Oslo University Hospital, Oslo, Norway; 6 School of Mathematical Sciences, University College Dublin, Belfield, Dublin, Ireland; 7 Lipids and Atherosclerosis Research Unit, Reina Sofía University Hospital, Maimonides Institute for Biomedical Research at Cordoba (IMIBIC), University of Cordoba, Ciber Phyisiopatology of Obesity and Nutrition (CIBEROBN), Instituto de Salud Carlos III, Cordoba, Spain; The Centre for Research and Technology, Hellas, Greece

## Abstract

Understanding the molecular link between diet and health is a key goal in nutritional systems biology. As an alternative to pathway analysis, we have developed a joint multivariate and network-based approach to analysis of a dataset of habitual dietary records, adipose tissue transcriptomics and comprehensive plasma marker profiles from human volunteers with the Metabolic Syndrome. With this approach we identified prominent co-expressed sub-networks in the global metabolic network, which showed correlated expression with habitual n-3 PUFA intake and urinary levels of the oxidative stress marker 8-iso-PGF_2α_. These sub-networks illustrated inherent cross-talk between distinct metabolic pathways, such as between triglyceride metabolism and production of lipid signalling molecules. In a parallel promoter analysis, we identified several adipogenic transcription factors as potential transcriptional regulators associated with habitual n-3 PUFA intake. Our results illustrate advantages of network-based analysis, and generate novel hypotheses on the transcriptomic link between habitual n-3 PUFA intake, adipose tissue function and oxidative stress.

## Author Summary

A fundamental goal in the field of nutritional genomics is defining the molecular link between diet and health. Human nutritional genomic studies are frequently hindered by a high level of unexplained variation in gene expression, protein and metabolite abundance, and clinical parameters – potentially attributable to variation in genotype, background diet, anthropometry, physical activity and health status. In our present study, relationships between adipose tissue gene expression, habitual diet and clinical markers of metabolic health were investigated in a cohort of individuals with impaired metabolic health, typical of the Metabolic Syndrome (MetS). Using multivariate statistics in conjunction with a novel approach to metabolic network analysis, we identified regions of the human metabolic network showing coordinated transcriptomic response to variation in n-3 PUFA intake and correlation with markers of metabolic health.

## Introduction

Dietary fat intake has profound effects on molecular processes of metabolic health. These effects are diverse and often subtle, representing a considerable analytical challenge in reaching system-level understanding. Transcriptomics has become a central technology in the development of molecular nutrition, having the capacity to produce expression data for every gene in a given genome. However, the major challenge is to apply appropriate techniques for extracting information from high-throughput datasets. Differentially expressed gene lists are an intuitive first choice, but they are hard to interpret in a biological context. Pathway analysis – typically implemented using gene set enrichment analysis – has become a standard method in the field of transcriptomic analysis [1], [2]. It is easy to implement and can simplify and contextualize large lists of differentially expressed genes, although this approach possesses technical limitations due to inherent redundancy among pathways and interconnectedness between one pathway and the next. Failure to appropriately account for these features can substantially limit biological interpretation of high-throughput datasets.

Network-level analysis has revealed detailed insight on metabolic regulation in type 2 diabetes and insulin resistance [3], [4]. Del Sol *et al.,* have proposed that in the emerging systems-level view of molecular biology, diseases should be viewed as a function of network perturbation rather than as isolated local changes [5]. Molecular networks may be classified in two categories: metabolic networks and protein interaction networks. Metabolic networks are inclusive, intuitive abstractions for representing system-level metabolism, as they incorporate all known metabolic interactions in a given species. Due to their size and complexity, however, they are analytically challenging. Previously applied analytical approaches include topological analysis (*e.g.,* identification of hub nodes and functional modules) [6] and reporter metabolite analysis [4].

A number of methods exist for analyzing transcriptomic data in the context of a global interaction network [7]. The majority of these methods focus on protein interaction networks, and aim to partition a global network into clusters/modules of genes, and identify clusters showing coordinated transcriptomic response. When modelling transcriptomic activity in metabolic networks, however, it is instructive to use path (rather than cluster) constructs, because paths match the native pattern of energy flux through a metabolic network. Cluster-based analysis of metabolic network activity performs well in identifying regions of a network with altered transcriptomic activity, but the identified clusters may contain disconnected sections of different paths of metabolite conversion. A given path of interest may thus be fractioned across several different neighbouring clusters, making it difficult to identify coordinated alteration of activity across that entire path. We therefore defined a method that identifies altered transcriptomic activity in the context of network paths rather than clusters. With this approach, we identified local coexpressed paths in the metabolic network showing covariance with recorded dietary intake of n-3 PUFA, and correlation with a urinary marker of oxidative stress. In a parallel analysis, investigation of the promoter regions of n-3 PUFA-correlated genes highlighted significantly over-represented binding sites for transcription factors related to adipogenesis.

## Materials and Methods

### Ethics statement

The LIPGENE human dietary intervention study was a randomized, controlled trial that complied with the 1983 Helsinki Declarations, approved by the local ethics committees of the 8 intervention centres (Dublin, Ireland; Reading, UK; Oslo, Norway; Marseille, France; Maastricht, The Netherlands; Cordoba, Spain; Krakow, Poland; Uppsala, Sweden). Written informed consent was attained from every participant as approved by each institutional ethical committee.

### Study design

The current study was conducted within the framework of the LIPGENE Integrated Project “Diet, genomics and the metabolic syndrome: an integrated nutrition, agro-food, social and economic analysis” (Clinical Trials. gov number: NCT00429195) and NuGO, The Nutrigenomics Organization (www.nugo.org); both European Union FP 6 initiatives. The subjects participated in the LIPGENE human dietary intervention study [8], although only baseline, pre-intervention samples were used in the present study. Samples were collected under standardised conditions according to a strict SOP [9]. Briefly, volunteers attended the clinics following a 12 h overnight fast; they were asked to abstain from alcohol, medications or vigorous exercise in the 24 h prior to assessment. For inclusion in the study, volunteers were required to be age 35–70 years, BMI 20–40 kg/m^3^ and show 3 or more of the following MetS criteria (based on slightly modified NCEP ATP-III): fasting plasma glucose 5.5–7.8 mmol/L, serum TAG ≥1.5 mmol/L, serum HDL-cholesterol <1.0 mmol/L in males, and <1.3 mmol/L in females, waist circumference >102 cm in males and >88 cm in females, and elevated blood pressure (systolic blood pressure ≥130 mmHg, diastolic blood pressure ≥85 mmHg or on prescribed blood pressure lowering medication). Habitual dietary intake was monitored for each volunteer by a 3 day weighed food dietary record, and assessed for daily intake of energy, carbohydrate, protein, fat, saturated fat (SFA), monounsaturated fat (MUFA), polyunsaturated fat (PUFA), and n-3 and n-6 PUFA [10]. Extensive metabolic profiling including plasma markers of inflammation, fatty acid pattern, plasma lipoproteins and apolipoprotein profiles, and markers of insulin sensitivity (Table 1), was performed as described by Tierney *et al.* [9]. Means and standard deviations for all dietary and plasma marker variables in our cohort are provided in supplementary Tables S1 and S2.

**Table 1:** Plasma markers measured in the LIPGENE study.

Fatty acid profile	Lipids	Apolipoproteins	IVGTT	Inflammatory markers
C14:0	Triglycerides	ApoA1	Glucose AUC*	C-Reactive protein
C16:	Cholesterol	ApoB		IL-6
C16:1	NEFA	ApoCII		TNFα
C18:0	TRL-TG	ApoCIII		sICAM
C18:1	TRL-C	ApoE		sVCAM
C18:2 n-6	LDL-C	TRL Apo B		Resistin
C18:3 n-6	T-HDL			Adiponectin
C18:4 n-3				PAI-1
C20:1				tPA
C20:3 n-6				Fibrinogen
C20:4 n-6				Leptin
C20:4 n-3				8-iso-PGF_2α_ (urinary)
C20:5 n-3				15-keto-PGF_2α_ (plasma)
C22: 4 n-6				
C22:5 n-3				
C22:6 n-3				

*Derived from relative area under the curve (AUC) of plasma glucose measurements (mmol/L) at 12 time points from 0 to 180 minutes following intravenous glucose challenge.

### Adipose tissue biopsy collection, RNA extraction and microarray hybridization

Subcutaneous adipose tissue samples were taken from the periumbilical area of 19 volunteers from the Norwegian and Spanish cohorts (10 female, 9 male) after an overnight fast. Needle biopsies were obtained after a 5 mm transdermal incision under local anaesthesia. Samples were rinsed in saline, put in RNA later and frozen immediately (−80°C) for subsequent analysis. Total RNA was extracted from adipose tissue using the RNeasy lipid tissue mini kit (Qiagen, U.K.). Briefly, 100 mg of adipose tissue was homogenised in Qiazol lysis reagent. After addition of chloroform, the homogenate was centrifuged to separate the aqueous and organic phases. Ethanol was added to the upper aqueous phase, and applied to the RNeasy spin column, where the total RNA was bound to the membrane, and phenol and other contaminants were washed away. RNA was then eluted in RNase-free water.

Extracted RNA was sent to ServiceXS (a high-throughput data service provider; www.servicexs.com) for labelling with the 3’ IVT express kit and hybridization to Affymetrix arrays. The microarray platform used in this study was custom designed by NuGO, and contained 16554 probe sets. This platform is designated ‘nugohs1a520180’, and we used the ‘entrezg’ version 12.1.0 annotation from the MBNI custom cdf database, reflecting the latest remapping of Affymetrix probes based on current data in the NCBI database (http://brainarray.mbni.med.umich.edu). Raw and GCRMA-normalized data are available from the Gene Expression Omnibus database, under accession GSE28070.

### Microarray QC and pre-processing

Raw microarray data were first assessed for quality using a set of standard QC tests, including array intensity distribution, positive and negative border element distribution, GAPDH and β-actin 3’/5’ ratios, centre of intensity and array-array correlation check. All QC tests were implemented in the R programming language (Version 2.11.1l, R Foundation for Statistical Computing), using the affyQCReport library. A batch effect was noted due to the arrays being hybridized on two separate days; thus, all subsequent analyses accounted for this effect by including batch number as a covariate in statistical models. It was also noted that the β-actin 3’/5’ ratios were higher than recommended (*i.e.,* greater than 3-fold intensity difference) in most samples, although this ratio has been shown to be higher when cRNA is synthesized using the Affymetrix 3’ IVT express kit, particularly with low input RNA quantities (http://media.affymetrix.com/support/technical/whitepapers/3_ivtexpresskit_whitepaper.pdf). Furthermore, all samples except 2 showed 3’/5’ GAPDH ratios within expected range of 1.25-fold, and no samples appeared suspect in RNA degradation plots. The 2 samples that did not meet the GAPDH ratio recommendation were removed from further analysis. All QC-verified samples were background corrected and normalized using the GCRMA normalization method, which accounts for nucleotide specific differences in hybridization efficiency. The normalized dataset was then filtered to remove genes with Mas5 ‘absent’ call on all arrays, and those showing the lowest 10% variance, resulting in a final dataset of 10618 genes.

### Statistical analysis of diet-gene and clinical marker-gene associations

Diet and plasma marker variables were first normalized with log or square root transformation as appropriate to reduce skewness and kurtosis. Sparse partial least squares regression (sPLS; [11]) and regularized canonical correlation analysis (rCCA; [12]) were used to assess relationships between dietary components and gene expression levels, and between clinical markers and gene expression levels, respectively. The mixOmics library of R functions was used to carry out the analysis [12]. Specifically, the *spls* function was used to fit the sPLS model, and the *network* function to produce the network of interactions. An sPLS model was fitted using dietary variables, sex, nationality and array batch number as predictors (sex, nationality and array batch number were included in order to identify and control for correlations between gene expression and these variables), and gene expression as response variables. Choice of PLS dimensions was determined using the *Q_h_^2^* variable previously proposed by Tenenhaus [13] which measures the relative contribution of each dimension *h* to the predictive power of the PLS model (see 11 and 13 for further details on sPLS and use of *Q_h_^2^*). With this approach, we retained 5 dimensions in the model, and retained all diet-gene pairs showing a similarity score >0.7 (using ‘threshold’ argument of the *network* function in the mixOmics library). This similarity score is a convention used in multivariate statistical methods; it ranges from 0 to 1, and corresponds to the distance between two given variables in the number of chosen dimensions [14].

The mixOmics library was used for rCCA modelling of plasma marker and gene expression data. The *rcc* function was used to define the canonical correlations and the canonical variates, *estim.regul* for estimation of regularization parameters and the *network* function to produce the network of interactions. In this case, datasets were not interpreted as predictors or responses given the more complex two-way relationship between plasma marker profile and tissue gene expression. Initial rCCA modelling including all plasma markers showed that correlations between gene expression and plasma fatty acid and lipid profile were so strong that they masked more subtle correlations between the remaining plasma markers and expression data. Consequently, separate rCCA analyses were performed: first, comparing adipose tissue gene expression to plasma fatty acids, lipids and apolipoprotein profile (including sex, nationality and array batch number as variables in the model); and second, comparing gene expression to plasma cytokines, IVGTT measurements, prostaglandin and urinary isoprostane (as before, including sex, nationality and array batch number as variables in the model). For comparison of gene expression vs. plasma fatty acids, lipids and apolipoproteins, the first 11 dimensions were retained in the model (as subsequent dimensions did not provide additional information to the model) and all gene-plasma marker pairs with a similarity score >0.75 were retained for subsequent analysis. Due to the very strong relationship between gene expression and plasma fatty acids, we observed that using a similarity score threshold of 0.7 resulted in a very high number of plasma marker-gene correlations (571 plasma marker-gene pairs passing threshold). Therefore, the higher threshold was chosen in this comparison in order to highlight only the strongest plasma marker-gene correlations, thereby facilitating downstream biological interpretation. For the second comparison (gene expression vs plasma cytokines, IVGTT measurements, prostaglandin and urinary isoprostane) the first 6 dimensions and all gene-plasma marker pairs with a similarity score >0.7, were retained in the model.

### Metabolic network analysis

We used the Edinburgh human metabolic network reconstruction [15]; (www.ehmn.bioinformatics.ed.ac.uk/), which contains reaction information for 1627 unique metabolites and 1371 unique metabolic enzymes. In its native form, this reconstruction is a metabolite-centred network (*i.e.,* nodes represent metabolites and edges are the enzymes that catalyze reactions between metabolites). For our analysis, the network was first transformed to an enzyme-centric construction (where 2 genes/proteins are linked if gene 1 produces a metabolite that is used as a metabolic substrate by gene 2). As is the norm in topology-based network analyses, we excluded currency metabolites (such as H_2_O, ATP and O_2_) from the network [16].

#### Assessment of gene-gene coexpression in the human metabolic network

Coexpression was assessed for each gene-gene pair in the metabolic network reconstruction using Akaike's information criterion (AIC), a criterion used to select an optimal model among competing possibilities [17]. As our network construction contains directionality information, coexpression between two genes could be assessed using a linear model of gene 2 expression as a function of gene 1 expression. In our human sample, however, we would expect the variables *sex* and *nationality* to be related to expression of some (but not all) genes in the dataset. The AIC approach allows selection of the optimal model among all possible combinations of predictor variables, thus including additional variables only where appropriate. The exact value of AIC is determined by 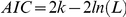
where *k* refers to the number of parameters in the statistical model, and L is the maximized likelihood value for the fitted model. An optimal model will have high likelihood while being parsimonious; thus, lower AIC values indicate a better model. As each gene pair in the network possesses directionality information (*i.e.,* gene 1 produces a compound that is metabolized by gene 2), expression level of gene 2 can be estimated as a function of gene 1 expression plus additional factors, *batch, sex* and *nationality: *

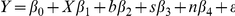
where *Y* and *X* represent expression of gene 2 and 1, respectively; *b*, *s* and *n* represent batch, sex and nationality; *β_0,_ β_1,_ β_2,_ β_3_* and *β_4_* represent the intercept and partial regression coefficients for each variable; and ε represents random error. We would expect a batch effect to be present across the entire platform; thus, this variable was included in all models. The AIC values were calculated for models containing *b* plus all possible combinations of the additional factors (*X, s* and *n*); gene 1 and gene 2 were considered to be coexpressed if gene 1 expression was present as a predictor variable in the optimal model.

### Extraction of diet-sensitive paths from the transcriptionally coordinated network

**Figure 1:**
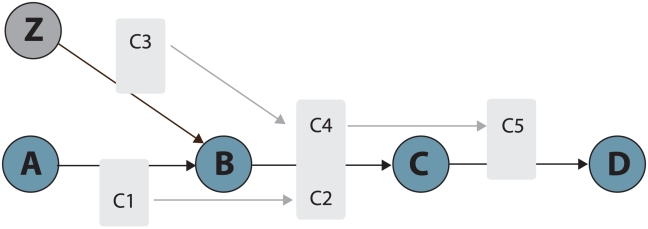
Assessing metabolic feasibility in network paths. The algorithm of network analysis in this study includes a two-step process: 1) extraction of connected paths from the node of interest to all others in the network; and 2) evaluation of metabolic feasibility of each candidate path. Given a candidate (*i.e.,* connected) path in the network through genes [A→B→C→D], the goal of the second step of the algorithm is to determine if a path of metabolite conversion can be traced from the first node to the last. In this simplified example, although a connectivity path can be traced from A to D, metabolite conversion cannot, emphasizing the importance of assessing metabolic feasibility in putative paths. A feasible path can only be traced from [Z→B→C→D] through conversion of metabolites [C3→C4→C5].

To identify paths of interest in the global interaction network, Dijkstra's shortest paths [18] were calculated from each diet-sensitive node to all others in the coexpressed subset of the global network (as determined above), taking into consideration directionality of node pair interactions. An algorithm was written in R to evaluate metabolic feasibility of each putative path – *i.e.* whether an unbroken path of metabolite conversion could be traced from one end to another (most recent scripts available on request). This concept of metabolic feasibility is an important consideration in analysis of global networks, because a connected path through the network does not necessarily indicate an unbroken path of metabolite conversion. Figure 1 illustrates the rationale behind metabolic feasibility (see supplementary Figure S1 for detailed description of the algorithm). The output of this algorithm is a list of feasible paths of metabolite conversion, wherein each path is strongly coexpressed (*i.e.,* between each gene pair in the path) and possesses a diet-correlated gene at the upstream end. To our knowledge, this is the first metabolic network analysis algorithm that explicitly considers metabolic feasibility and adjacent pair-wise coexpression in analysis of network paths. This represents an informative alternative to network clustering analysis.

### Promoter analysis

The TFM-explorer tool [19] was used to identify significantly over-represented transcription factor binding sites (TFBSs) among genes with expression showing positive correlation with n-3-PUFA intake. Using the promoter regions spanning -2000+200 bp relative to the transcription start site and all vertebrate transcription factor matrices from the Jaspar database (jaspar.cgb.ki.se), TFM-explorer returned all TFBSs that were significantly over-represented at a level of p<0.0001.

## Results

### Multivariate analysis identifies a strong relationship between dietary n-3 PUFA, adipose tissue gene expression and markers of metabolic health

**Figure 2:**
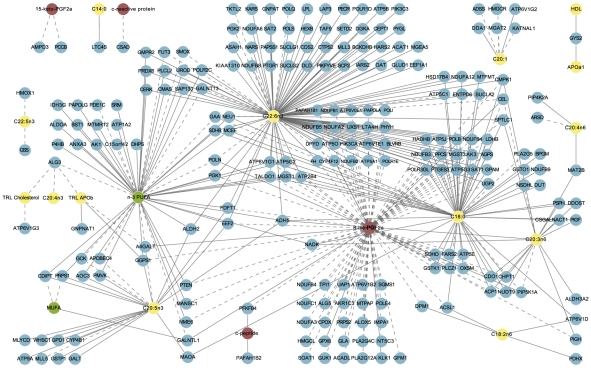
Network of associations between dietary intake, adipose gene expression, and phenotypic markers, determined by sPLS and rCCA. Green nodes: dietary variables; yellow: lipid, fatty acid and apolipoprotein variables; red: inflammatory and oxidative stress markers; blue: genes (enzymes). Solid lines: positive correlation (rCCA)/covariance (sPLS); dashed lines: negative correlation/covariance.

Results from sPLS indicated that among all dietary variables, the registered dietary intake of n-3-PUFA showed the strongest covariance with adipose tissue gene expression. Of the 53 n-3-PUFA-correlated genes identified in the sPLS analysis, 41 positively correlated and 12 negatively correlated with n-3-PUFA intake (Figure 2; Supplementary Table S3). Dietary intake of MUFA also showed strong covariance with expression of three of these genes (one positive: *GALNTL1*; two negative: *CDIPT, PRPS1*). It was also noted that the expression level of these three genes covaried in opposing direction with intake of n-3-PUFA and MUFA, reflecting the inverse relationship between habitual dietary consumption of these two dietary fatty acids in our population. To assess if > = 53 n-3 PUFA-correlated genes would be detected by chance alone, we permuted the sample labels and re-ran the sPLS analysis 100 times. These permutation tests yielded an average of 2.38 and median of 0 n-3 PUFA-correlated genes, suggesting that the 53 genes identified in our original dataset were unlikely to be identified by chance alone.

rCCA results showed that among the measured plasma lipids, fatty acids and apolipoproteins, plasma DHA, stearic acid and EPA correlated most strongly with adipose tissue gene expression (Figure 2; Supplementary Table S4). At the chosen threshold of 0.75, DHA [C22:6 n-3] correlated with the expression of 113 genes, followed by plasma stearic acid [C18:0]: 60 genes, and EPA [C20:5 n-3]: 21 genes. Comparison of sPLS and rCCA results highlighted 26 genes that were related to dietary n-3-PUFA intake as well as plasma DHA levels, reflecting the expected correlation between dietary fat intake and plasma fatty acid profile [20]. Among markers of inflammation, oxidative stress and insulin resistance, urinary 8-iso-PGF_2α_ correlated most strongly with adipose gene expression, resulting in 96 gene correlations, 48 of which also correlated with plasma DHA (Figure 2; Supplementary Table S5). Any variables not included in Figure 2 (*e.g.,* IVGTT) did not correlate with any adipose tissue genes at the chosen threshold.

The complete metabolic network included 1371 nodes and 65637 directed edges; the transcriptionally coexpressed (TC) subset contained 602 nodes and 5414 directed edges (supplementary Figure S2). To identify the biological functions predominant in this network, the largest connected subset of the TC subset network was partitioned into topological modules using a simulated annealing approach [21], as implemented by the *spinglass.community* function in the igraph library in R. This modular partitioning identified 3 topological modules. Hypergeometric tests were performed using the Category library in R to identify significantly over-represented gene ontology (www.geneontology.org) ‘biological process’ terms in each module. Briefly, over-represented terms in the first module related primarily to phosphatidylinositol and lipid metabolism; those in second related to nucleic acid metabolism; and the third module was more heterogeneous, consisting of cellular ketone metabolism, red-ox processes, and lipid and protein catabolism terms (see supplementary Table S6 for expanded results).

### Coexpressed paths in the metabolic network related to n-3 PUFA intake

**Figure 3:**
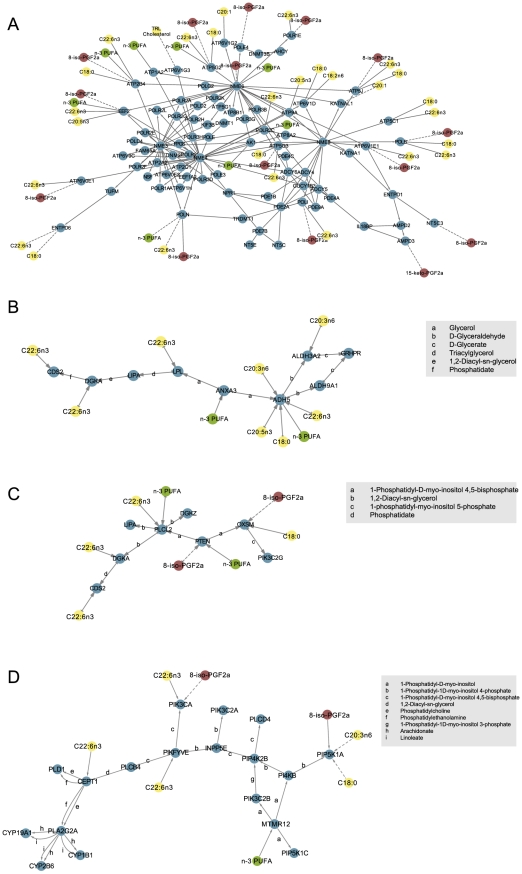
Transcriptionally coordinated paths leading from genes correlated with habitual n-3 PUFA intake. Green nodes: dietary variables; yellow: lipid, fatty acid and apolipoprotein variables; red: inflammatory and oxidative stress markers; blue: genes (enzymes). Dashed lines indicate negative correlation. A: Path linked to *AK1*; B: Detailed path linked to *ANXA3*; C: Detailed path linked to *PTEN*; D: Detailed path linked to *MTMR12*.

Diet-sensitive path extraction from the TC network revealed 755 unique paths greater than length 2 originating from 30 n-3 PUFA-sensitive genes, although paths leading from each diet-sensitive gene collapsed into tree-like structures (Figure 3). The most complex n-3 PUFA-sensitive path (in terms of path size and link density) centred on the *AK1* gene (Figure 3A). The genes in the *AK1* path are mostly involved in the highly redundant processes of energy and nucleotide metabolism, explaining the high link density in this region of the network. The majority of metabolic links in the *AK1* path are different nucleotides and energy metabolism cofactors such as ATP, ADP and AMP. These metabolites are normally classified as currency metabolites and were removed from the rest of the network, although they were retained in this region where they act as primary reactants and products. Of the nodes in this path, an additional 7 correlated with dietary intake of n-3 PUFA, 28 strongly correlated with plasma fatty acid levels, and 14 with urinary 8-iso-PGF_2α_, suggesting that activity in this region of the metabolic network is sensitive to dietary intake of n-3 PUFA and correlated with metabolic health.

The *ANXA3*, *PTEN* and *MTMR12*-linked paths (Figure 3B-D) are interesting from a biological perspective because they each incorporate elements of lipid metabolism. The *ANXA3-*linked path primarily includes reactions involved in metabolism of glycerolipids, glycerophospholipids, arachidonic acid (AA) and linoleic acid (LA). *ANXA3* is connected to *ADH5* and *LPL* via the glycerol metabolite, which is further metabolized by *LIPA* and *DGKA* to form 1,2-diacyl-sn-glycerol (1,2-diacylglycerol) and phosphatidate on one branch of the path, and by *ALDH* isoforms to form d-glyceraldehyde and d-glycerate on the other. Five genes in this path correlated strongly with plasma levels of DHA, stearic acid, dihomo-gamma-linolenic acid and/or EPA.

The *PTEN-*linked path includes reactions that metabolize inositol phosphate- and lipid-related metabolites. *PTEN* is linked to *OXSM* and *PLCL2* via the 1-phosphatidyl-D-myo-inositol 4,5-bisphosphate, which is metabolized by these enzymes to form 1-phosphatidyl-myo-inositol 5-phosphate and 1,2-diacyl-sn-glycerol. This 1,2-diacyl-sn-glycerol is further metabolized by *DGKA* to form phosphatidate. Two genes in this path – *PTEN* and *OXSM –* were inversely correlated with urinary 8-iso-PGF_2α_, and three –*PLCL2, DGKA* and *CDS2* – were positively correlated with plasma DHA level.

The *MTMR12* path (Figure 3D) is also linked to lipid metabolism via phosphatidylcholine, through a more complex upstream path involving inositol phosphate derivatives. At the downstream end of this path, cytochrome p450 enzymes (*MYP19A1, CYP2B6* and *CYP1B1*) act on AA and LA as substrates to form diverse epoxyeicosatrienoic acids (EET), hydroxyeicosatrienoic acids (HETE) and epoxyoctadecenoic acids (EpOME), involved in the resolution of inflammation with subsequent relevance to cardiovascular disease [22], [23]. Two genes in this path – *PIK3CA*and *PIP5K1A* – were negatively correlated with urinary 8-iso-PGF_2α_; *PIKFYVE, PIK3CA,* and *CEPT1* were positively correlated with plasma DHA, and *PIP5K1A* with plasma stearic acid and dihomo-gamma-linolenic acid.

To assess whether a similar group of paths would be extracted from any TC network – *e.g.,* due to higher connectivity in certain regions of the network – we generated such a TC network from publicly available muscle tissue microarray data from obese individuals (GEO accession: GSE474), and extracted paths leading from the n-3 PUFA-sensitive genes identified in the present study. In this muscle tissue TC network we found only 24 paths of maximum length three leading from ten of the n-3-PUFA-sensitive genes (supplementary Table S7). Furthermore, these paths did not intersect to form a larger sub-network.

To compare our network analysis with a standard approach to pathway analysis, hypergeometric tests were performed to identify KEGG pathways significantly enriched (using the *hyperGTest* function in the R ‘Category’ library) for the n-3PUFA-sensitive genes identified in our sPLS analysis. This analysis returned four KEGG pathways greater than length four (Table 2). Of these pathways, the top three - biosynthesis of plant hormones, biosynthesis of terpenoids and steroids and biosynthesis of alkaloids derived from terpenoid and polyketide - have 45 genes in common. Accordingly, the same 7 n-3 PUFA-sensitive genes (*PMVK*, *FDFT1*, *ALDOA*, *IDH3G*, *PGK1*, *SDHB*, *GGPS1*) were present in each pathway. Thus, the apparent enrichment of the biosynthetic plant hormones pathway is probably an artifact of the high degree of overlap with other pathways in the database. Closer inspection of these pathways in the KEGG database showed that they are large, diverse and disjointed pathways, including many parallel processes. For example, the pathway for biosynthesis of terpenoids and steroids includes of subsets of glycolysis, limonene and pinene degradation, terpenoid backbone biosynthesis, carotenoid biosynthesis and geraniol degradation. The n-3 PUFA-correlated genes were distributed across these processes rather than occurring in a single one.

**Table 2:** KEGG pathways differentially regulated by n-3 PUFA intake using a hypergeometric test.

Term	Expected count	Observed count	Pathway size	P value
Biosynthesis of plant hormones	3.607	9	60	0.007
Biosynthesis of terpenoids and steroids	2.886	7	48	0.020
Biosynthesis of alkaloids derived from terpenoid and polyketide	3.066	7	51	0.028
3-Chloroacrylic acid degradation	0.361	2	6	0.046

### Promoter analysis highlights over-represented adipogenic transcription factors among n-3 PUFA-correlated genes

**Figure 4:**
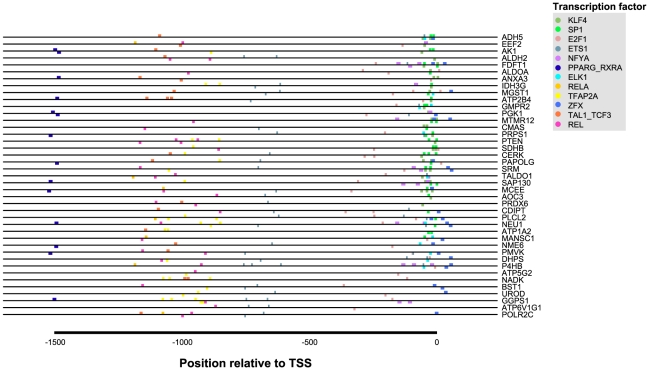
Significantly over-represented transcription factor binding sites in promoter regions of genes correlated with habitual n-3 PUFA intake. The promoter region of each gene is depicted, with coloured boxes denoting binding site location(s) of transcription factors displayed at right. TSS: transcription start site.

Figure 4 illustrates over-represented TFBSs among genes showing positive correlation with the intake of n-3 PUFA (from our sPLS results). The three most significantly over-represented TFBSs were those of Krüppel-like factor 4 (KLF4), specificity protein 1 (SP1) and E2F1 transcription factors. KLF4 is involved in adipogenesis, specifically by binding to the promoter of the *CEBPB* (C/EBPβ) gene [24]. *CEBPB* was not present in the Edinburgh human metabolic network (as it is not a metabolic enzyme). Thus, an expanded sPLS analysis was performed, comparing dietary intake to all genes on the Affymetrix microarray. This analysis identified *CEBPB* expression to be positively correlated with n-3 PUFA intake (data not shown). Although no additional adipogenic genes were identified at this correlation threshold of 0.7, reducing the threshold to 0.6 revealed a number of additional adipogenic factors showing positive correlation with n-3 PUFA intake – including *ADIPOQ, BMP2, CFD, FABP4, LIPE, LPL* and *PLIN.*

SP1 is a broadly acting transcription factor operating in conjunction with NF-YA, SREBP and PPARγ in promoting lipogenesis. The NF-YA and PPARγ TFBSs were also significantly over-represented in our group of n-3 PUFA-sensitive genes, although SREBP TFBS was not. E2F1 is a transcription factor involved in early adipogenesis, and positively regulates transcription of PPARγ [25]. Interestingly, all but one PPARγ TFBS-containing genes in our sample also contained an E2F1 TFBS.

## Discussion

An emerging limitation to pathway analysis of transcriptomic data is that documented pathway models tend to overlap and intersect, yielding analytical results that are biased, incomplete or both [26]. Our network approach did not segregate metabolic processes into discrete pathway models, thereby revealing inherent overlaps and intersections between pathways. Examples of this pattern are seen in the *ANXA3, PTEN* and *MTMR12* paths, which incorporate connected reactions from the metabolic pathways of inositol phosphate derivatives, glycerolipids, glycerophospholipids, arachidonic and linoleic acid. Intersections of these pathways are clear when viewed in the network context, but less evident when each canonical pathway is assessed separately. Cross-talk between metabolism of lipids/fatty acids and inositol phosphate derivatives plays an important role in the induction of signalling cascades by dietary and fat [27]. Figure 3B-D illustrates paths of triglyceride metabolism including formation of intermediate metabolites such as diacylglycerol and phosphatidate. These metabolites act as signalling molecules that affect a wide range of cellular functions like insulin signalling, inflammation, cellular differentiation and proliferation and oxidative stress [28], [29]. Thus, it is of particular interest that genes in the *PTEN* and *MTMR12* paths show strong inverse correlation with urinary 8-iso-PGF_2α_ – a marker of systemic oxidative stress.

Previous work has described an inverse relationship between n-3 PUFA intake and n-6 fatty acid-derived prostaglandins (*e.g.,* PGF_2α_) in the plasma of Alzheimer's disease patients [30], urine of healthy males [31] and plasma and urine of dyslipidaemic and type 2 diabetic individuals [32]. A prevalent hypothesis for this relationship is that n-3 and n-6 PUFA compete for the same enzyme systems, and consequently, increased n-3 PUFA intake precludes production of n-6 fatty acid-derived prostaglandins [33]. In addition, n-3 PUFA may exert independent anti-inflammatory effects through unique receptors and enzyme systems, regardless of n-6 fatty acid intake [34]. Our analysis identified 17 genes showing opposing direction of correlation with n-3 PUFA intake and urinary 8-iso-PGF_2α_ (Figure 2). Furthermore, results from network analysis identified precise coexpressed regions of the metabolic network showing positive correlation with dietary n-3 PUFA intake and plasma DHA, and negative correlation with urinary 8-iso-PGF_2α_ in the cohort of MetS subjects (Figure 3). The sub-network illustrated in Figure 3A is interesting in this context because it contains numerous members of the electron transport chain, including 20 isoforms of ATPase/ATP synthase, six of which were negatively correlated with 8-iso-PGF_2α_. Further investigation of diet-dependent energy flux through these network regions may provide insight on the precise relationship between n-3 PUFA intake and adipose tissue oxidative stress. Comparing n-3 PUFA intake directly to urinary 8-iso-PGF_2α_ resulted in only a near-significant trend (p = 0.077; p = 0.0828 after adjusting for sex); a recent publication of findings from the larger LIPGENE cohort reported a similar near-significant trend [35]. This may be due to the number of molecular intermediates between dietary intake and urinary output, highlighting the increased clarity provided by analysis of tissue-level high throughput data in the framework of a global metabolic network.

To understand the potential regulatory consequences of dietary n-3 PUFA intake on adipose tissue biology, we analysed the promoter regions of n-3 PUFA-correlated genes to identify significantly over-represented transcription factor binding sites. Results from this analysis highlighted significantly over-represented transcription factors related to adipogenesis. The most strongly over-represented transcription factors were KLF4, SP1 and E2F1. SP1 and KLF4 share similar GC-rich target binding sites [36], which is evident in their overlapping binding sites in Figure 4. Although limited work has focused on joint activity of these transcription factors in adipose tissue, KLF4 has been shown to inhibit SP1 activity in the gut by competitive TFBS binding [37]. PPARγ was also identified as significantly over-represented among genes correlated with habitual n-3 PUFA intake. PPARγ is arguably the most well-studied transcription factor in the field of diet-related transcriptomic regulation, and is the subject of many reviews on the subject [38], [39], [40], [41]. In addition to its role as a central regulator of adipogenesis, lipid storage and combustion, PPARγ also protects against oxidative stress and inflammation [42]. Dietary n-3 PUFA are potent inducers of PPARγ expression in adipocytes [43] and preadipocytes [44]. Accordingly, n-3 PUFA supplementation has yielded positive effects on weight gain in cancer [45] and Alzheimer's patients [46], and reduced lipotoxicity in a range of experimental models [47]. Future work should clarify the contribution of these adipogenic transcription factors to adipose tissue function, particularly given the positive correlation in our present study between dietary n-3 PUFA intake and expression of additional adipogenic genes, including *CEBPB, ADIPOQ, BMP2, CFD, FABP4, LIPE, LPL* and *PLIN1.*

In conclusion, we have taken a joint multivariate and network-based approach to transcriptomic analysis, relying on known metabolic reaction information to reveal coordinated paths of metabolite conversion. This approach highlighted coexpressed regions of the metabolic network with opposing direction of correlation with habitual n-3 PUFA intake and urinary isoprotane levels - relationships that were not identified using a traditional pathway enrichment test. Promoter analysis further highlighted adipogenic transcription factors as potential transcriptional regulators of n-3 PUFA-correlated genes.

## Supporting Information

Figure S1 Schematic illustration of pathway extraction algorithm; data frame at right shows example network data file at each step of algorithm. The key goal of this algorithm is to assess a linked path of nodes (in this case A→B→C→D), to identify if an unbroken path of metabolite conversion can be traced from the first node to the last. Given a node of interest (A) and a network path leading from A (as determined by Djikstra's algorithm; step 1) the algorithm examines each reaction pair in sequence, starting with the pair linked to the node of interest (step 2). The total list of interactions from A→B, and B→C are extracted from network file (step 3). Metabolites (and associated reactions) are removed from the pair of reactions if they cannot be associated with a path of conversion linking reaction 1 (A→B) to reaction 2 (B→C) (step 4). Self-linked reactions are included – e.g., B→B, where node B produces a metabolite through interaction with A, and converts the same metabolite to a different one that is further metabolized by node C (the algorithm only considers a single self-linked loop within each reaction pair, if present). If an unbroken path remains after removal of extraneous metabolites (step 4a), reaction pair is valid and algorithm continues to next pair of reactions (step 5). If not (step 4b), function exits.
(EPS)

Figure S2 Diet-gene and phenotype-gene relationships, and modular partitioning mapped to the transcriptionally coordinated human metabolic network. Green nodes: dietary variables; yellow: lipid, fatty acid and apolipoprotein variables; red: inflammatory and oxidative stress markers; blue: genes (enzymes). Enzyme nodes are connected if they meet two conditions: enzyme 1 produces a metabolite that is metabolized by enzyme 2, and genes encoding enzymes 1 and 2 show positive coexpression in the adipose tissue transcriptomic data. Dashed lines connecting diet-gene and plasma marker-gene pairs indicate negative correlation. Node shape indicates assignment in the 3 primary topological modules. Diamond: module 1; triangle: module 2; square: module 3.
(EPS)

Table S1 Summary of anthropometric characteristics and habitual dietary patterns in the LIPGENE transcriptomic study cohort.
(DOCX)

Table S2 Summary of plasma and urinary markers of metabolic health in the LIPGENE transcriptomic study cohort.
(DOCX)

Table S3 Results from sPLS of adipose tissue gene expression and components of recorded habitual diet. Diet-gene pairs passing the similarity threshold of 0.7 are shown.
(DOCX)

Table S4 Results from rCCA of adipose tissue gene expression and plasma fatty acids, lipids and apolipoproteins. Plasma marker-gene pairs passing the similarity threshold of 0.75 are shown.
(DOCX)

Table S5 Results from rCCA of adipose tissue gene expression and plasma cytokines, IVGTT measurements, prostaglandin and urinary isoprostane. Plasma marker-gene pairs passing the similarity threshold of 0.7 are shown.
(DOCX)

Table S6 Significantly overrepresented Gene Ontology ‘biological process’ terms in the adipose tissue TC network modules. Top 10 terms for each module are shown.
(DOCX)

Table S7 Paths detected by applying network analysis algorithm to test muscle tissue dataset (GEO accession GSE474).
(DOCX)
