# Supplementary material for: Transcriptomic Coordination in the Human Metabolic Network Reveals Links between n-3 Fat Intake, Adipose Tissue Gene Expression and Metabolic Health
Source: PLoS Comput Biol. 2011 Nov 3;7(11):e1002223. doi: 10.1371/journal.pcbi.1002223 (PMC3207936; doi:10.1371/journal.pcbi.1002223)
Supplement: Table S1 — Summary of anthropometric characteristics and habitual dietary patterns in the LIPGENE transcriptomic study cohort. (DOCX) [file pcbi.1002223.s003.docx]

**Supplementary Table S1.** Summary of anthropometric characteristics and habitual dietary patterns in the LIPGENE transcriptomic study cohort.

| **Variable** | **Mean** | **Standard deviation** |
| --- | --- | --- |
| Nationality (Norway; Spain) | (N=11; S=6) | - |
| Age | 58 | 8.276 |
| Sex | (F=9; M=8) | - |
| Body weight (kg) | 92.318 | 11.634 |
| Basal Metabolic Rate (MJ/day) | 7.199 | 1.095 |
| Total Energy intake (MJ/day) | 8.127 | 1.957 |
| Total fat (g/day) | 36.206 | 6.998 |
| SAFA (% total fat intake) | 12.212 | 3.054 |
| MUFA (%) | 14.318 | 4.617 |
| PUFA (%) | 5.629 | 1.465 |
| n-3 PUFA (g/day) | 1.621 | 0.895 |
| n-6 PUFA (g/day) | 10.235 | 4.333 |
| Total carbohydrates (g/day) | 45.229 | 7.512 |
| Protein (g/day) | 16.918 | 2.891 |
