# Supplementary material for: Transcriptomic Coordination in the Human Metabolic Network Reveals Links between n-3 Fat Intake, Adipose Tissue Gene Expression and Metabolic Health
Source: PLoS Comput Biol. 2011 Nov 3;7(11):e1002223. doi: 10.1371/journal.pcbi.1002223 (PMC3207936; doi:10.1371/journal.pcbi.1002223)
Supplement: Table S2 — Summary of plasma and urinary markers of metabolic health in the LIPGENE transcriptomic study cohort. (DOCX) [file pcbi.1002223.s004.docx]

**Supplementary Table S2.** Summary of plasma and urinary markers of metabolic health in the LIPGENE transcriptomic study cohort.

| **Variable** | **Mean** | **Standard deviation** |
| --- | --- | --- |
| apoa1 (g/L) | 1.403 | 0.2475 |
| apob (g/L) | 1.074 | 0.1558 |
| apocii (mg/L) | 47.776 | 13.4386 |
| apociii (mg/L) | 154.171 | 28.5549 |
| TRLapob (mg/L) | 50.683 | 45.7528 |
| C14:0 (mg/mL) | 0.039 | 0.0236 |
| C16:0 (mg/mL) | 0.549 | 0.1893 |
| C16:1 (mg/mL) | 0.025 | 0.0200 |
| C18:0 (mg/mL) | 0.078 | 0.0379 |
| C18:1 (mg/mL) | 0.543 | 0.1638 |
| C18:2 (n-6) (mg/mL) | 0.623 | 0.1836 |
| C18:3 (n-6) (mg/mL) | 0.001 | 0.0006 |
| C18:4 (n-3) (mg/mL) | 0.001 | 0.0011 |
| C20:1 (mg/mL) | 0.003 | 0.0028 |
| C20:3 (n-6) (mg/mL) | 0.019 | 0.0114 |
| C20:4 (n-6) (mg/mL) | 0.142 | 0.0467 |
| C20:4 (n-3) (mg/mL) | 0.001 | 0.0008 |
| C20:5 (n-3) (mg/mL) | 0.032 | 0.0282 |
| C22:4 (n-6) (mg/mL) | 0.003 | 0.0026 |
| C22:5 (n-3) (mg/mL) | 0.011 | 0.0036 |
| C22:6 (n-3) (mg/mL) | 0.064 | 0.0322 |
| cpeptide (ng/ml) | 3.116 | 1.0563 |
| crp (mg/ml) | 6.479 | 3.8679 |
| il6 (pg/ml) | 5.170 | 4.7063 |
| tnfa (pg/ml) | 6.532 | 2.3275 |
| sicam (ng/ml) | 279.419 | 44.1521 |
| svcam (ng/ml) | 615.292 | 209.0874 |
| adiponectin (ug/ml) | 3.372 | 1.9346 |
| pai1 (ng/ml) | 52.158 | 31.5843 |
| tpa (ng/ml) | 11.535 | 5.6242 |
| fibrinogen (mg/ml) | 309.567 | 64.3704 |
| leptin (ng/ml) | 25.829 | 26.7141 |
| TAG (mmol/L) | 1.715 | 0.6193 |
| CHOL (mmol/L) | 5.603 | 0.6256 |
| NEFA umol.l | 681.120 | 252.6180 |
| TRLTG (mmol/L) | 0.783 | 0.4364 |
| TRLC (mmol/L) | 0.361 | 0.2333 |
| LDLC (mmol/L) | 3.416 | 0.8256 |
| THDL (mmol/L) | 1.093 | 0.2317 |
| 15-keto-PGF2a (mmol/mmol creatinine) | 0.132 | 0.0434 |
| 8-iso-PGF2a (mmol/mmol creatinine) | 0.436 | 0.1046 |
