# Supplementary material for: Transcriptomic Coordination in the Human Metabolic Network Reveals Links between n-3 Fat Intake, Adipose Tissue Gene Expression and Metabolic Health
Source: PLoS Comput Biol. 2011 Nov 3;7(11):e1002223. doi: 10.1371/journal.pcbi.1002223 (PMC3207936; doi:10.1371/journal.pcbi.1002223)
Supplement: Table S3 — Results from sPLS of adipose tissue gene expression and components of recorded habitual diet. Diet-gene pairs passing the similarity threshold of 0.7 are shown. (DOCX) [file pcbi.1002223.s005.docx]

**Supplementary Table S3.** Results from sPLS of adipose tissue gene expression and components of recorded habitual diet. Diet-gene pairs passing the similarity threshold of 0.7 are shown.

| **Dietary variable** | **Gene symbol** | **Gene name** | **Similarity score** |
| --- | --- | --- | --- |
| MUFA (% total fat intake) | *CDIPT* | *CDP-diacylglycerol--inositol 3-phosphatidyltransferase* | -0.72 |
| MUFA (% total fat intake) | *PRPS1* | *phosphoribosyl pyrophosphate synthetase 1* | -0.78 |
| MUFA (% total fat intake) | *GALNTL1* | *UDP-N-acetyl-alpha-D-galactosamine:polypeptide N-acetylgalactosaminyltransferase-like 1* | 0.7 |
| n-3 PUFA (g/day) | *ALG3* | *asparagine-linked glycosylation 3, alpha-1,3- mannosyltransferase homolog (S. cerevisiae)* | -0.75 |
| n-3 PUFA (g/day) | *NME6* | *non-metastatic cells 6, protein expressed in (nucleoside-diphosphate kinase)* | 0.71 |
| n-3 PUFA (g/day) | *CDIPT* | *CDP-diacylglycerol--inositol 3-phosphatidyltransferase* | 0.8 |
| n-3 PUFA (g/day) | *PMVK* | *phosphomevalonate kinase* | 0.72 |
| n-3 PUFA (g/day) | *GALNT13* | *UDP-N-acetyl-alpha-D-galactosamine:polypeptide N-acetylgalactosaminyltransferase 13 (GalNAc-T13)* | -0.71 |
| n-3 PUFA (g/day) | *ADH5* | *alcohol dehydrogenase 5 (class III), chi polypeptide* | 0.74 |
| n-3 PUFA (g/day) | *DHPS* | *deoxyhypusine synthase* | 0.7 |
| n-3 PUFA (g/day) | *EEF2* | *eukaryotic translation elongation factor 2* | 0.72 |
| n-3 PUFA (g/day) | *AK1* | *adenylate kinase 1* | 0.7 |
| n-3 PUFA (g/day) | *ALDH2* | *aldehyde dehydrogenase 2 family (mitochondrial)* | 0.72 |
| n-3 PUFA (g/day) | *FDFT1* | *farnesyl-diphosphate farnesyltransferase 1* | 0.79 |
| n-3 PUFA (g/day) | *ALDOA* | *aldolase A, fructose-bisphosphate* | 0.71 |
| n-3 PUFA (g/day) | *PLCL2* | *phospholipase C-like 2* | 0.77 |
| n-3 PUFA (g/day) | *FUT3* | *fucosyltransferase 3 (galactoside 3(4)-L-fucosyltransferase, Lewis blood group)* | -0.7 |
| n-3 PUFA (g/day) | *GAA* | *glucosidase, alpha; acid* | -0.74 |
| n-3 PUFA (g/day) | *GCK* | *glucokinase (hexokinase 4)* | -0.7 |
| n-3 PUFA (g/day) | *ANXA3* | *annexin A3* | 0.72 |
| n-3 PUFA (g/day) | *IDH3G* | *isocitrate dehydrogenase 3 (NAD+) gamma* | 0.71 |
| n-3 PUFA (g/day) | *POLN* | *polymerase (DNA directed) nu* | -0.74 |
| n-3 PUFA (g/day) | *APOBEC4* | *apolipoprotein B mRNA editing enzyme, catalytic polypeptide-like 4 (putative)* | -0.7 |
| n-3 PUFA (g/day) | *MGST1* | *microsomal glutathione S-transferase 1* | 0.73 |
| n-3 PUFA (g/day) | *NEU1* | *sialidase 1 (lysosomal sialidase)* | 0.71 |
| n-3 PUFA (g/day) | *ATP1A2* | *ATPase, Na+/K+ transporting, alpha 2 polypeptide* | 0.72 |
| n-3 PUFA (g/day) | *ATP2B4* | *ATPase, Ca++ transporting, plasma membrane 4* | 0.71 |
| n-3 PUFA (g/day) | *P4HB* | *prolyl 4-hydroxylase, beta polypeptide* | 0.75 |
| n-3 PUFA (g/day) | *GMPR2* | *guanosine monophosphate reductase 2* | 0.76 |
| n-3 PUFA (g/day) | *PDE1C* | *phosphodiesterase 1C, calmodulin-dependent 70kDa* | -0.74 |
| n-3 PUFA (g/day) | *ATP5G2* | *ATP synthase, H+ transporting, mitochondrial Fo complex, subunit C2 (subunit 9)* | 0.73 |
| n-3 PUFA (g/day) | *PGK1* | *phosphoglycerate kinase 1* | 0.72 |
| n-3 PUFA (g/day) | *A4GALT* | *alpha 1,4-galactosyltransferase* | -0.75 |
| n-3 PUFA (g/day) | *POLR2C* | *polymerase (RNA) II (DNA directed) polypeptide C, 33kDa* | 0.78 |
| n-3 PUFA (g/day) | *SMOX* | *spermine oxidase* | -0.73 |
| n-3 PUFA (g/day) | *MTMR12* | *myotubularin related protein 12* | 0.72 |
| n-3 PUFA (g/day) | *MANSC1* | *MANSC domain containing 1* | 0.71 |
| n-3 PUFA (g/day) | *CMAS* | *cytidine monophosphate N-acetylneuraminic acid synthetase* | 0.7 |
| n-3 PUFA (g/day) | *PRPS1* | *phosphoribosyl pyrophosphate synthetase 1* | 0.77 |
| n-3 PUFA (g/day) | *PTEN* | *phosphatase and tensin homolog* | 0.71 |
| n-3 PUFA (g/day) | *GALNTL1* | *UDP-N-acetyl-alpha-D-galactosamine:polypeptide N-acetylgalactosaminyltransferase-like 1* | -0.81 |
| n-3 PUFA (g/day) | *SDHB* | *succinate dehydrogenase complex, subunit B, iron sulfur (Ip)* | 0.7 |
| n-3 PUFA (g/day) | *CERK* | *ceramide kinase* | 0.7 |
| n-3 PUFA (g/day) | *PAPOLG* | *poly(A) polymerase gamma* | 0.73 |
| n-3 PUFA (g/day) | *NADK* | *NAD kinase* | 0.74 |
| n-3 PUFA (g/day) | *SRM* | *spermidine synthase* | 0.75 |
| n-3 PUFA (g/day) | *BST1* | *bone marrow stromal cell antigen 1* | 0.75 |
| n-3 PUFA (g/day) | *TALDO1* | *transaldolase 1* | 0.74 |
| n-3 PUFA (g/day) | *UROD* | *uroporphyrinogen decarboxylase* | 0.78 |
| n-3 PUFA (g/day) | *SAP130* | *Sin3A-associated protein, 130kDa* | 0.72 |
| n-3 PUFA (g/day) | *MCEE* | *methylmalonyl CoA epimerase* | 0.73 |
| n-3 PUFA (g/day) | *AOC3* | *amine oxidase, copper containing 3 (vascular adhesion protein 1)* | 0.75 |
| n-3 PUFA (g/day) | *C15orf42* | *chromosome 15 open reading frame 42* | -0.75 |
| n-3 PUFA (g/day) | *GGPS1* | *geranylgeranyl diphosphate synthase 1* | 0.71 |
| n-3 PUFA (g/day) | *ATP6V1G1* | *ATPase, H+ transporting, lysosomal 13kDa, V1 subunit G1* | 0.7 |
| n-3 PUFA (g/day) | *PRDX6* | *peroxiredoxin 6* | 0.73 |
