# Supplementary material for: Transcriptomic Coordination in the Human Metabolic Network Reveals Links between n-3 Fat Intake, Adipose Tissue Gene Expression and Metabolic Health
Source: PLoS Comput Biol. 2011 Nov 3;7(11):e1002223. doi: 10.1371/journal.pcbi.1002223 (PMC3207936; doi:10.1371/journal.pcbi.1002223)
Supplement: Table S4 — Results from rCCA of adipose tissue gene expression and plasma fatty acids, lipids and apolipoproteins. Plasma marker-gene pairs passing the similarity threshold of 0.75 are shown. (DOCX) [file pcbi.1002223.s006.docx]

**Supplementary Table S4.** Results from rCCA of adipose tissue gene expression and plasma fatty acids, lipids and apolipoproteins. Plasma marker-gene pairs passing the similarity threshold of 0.75 are shown.

| **Plasma marker** | **Gene** | **Gene name** | **Similarity score** |
| --- | --- | --- | --- |
| apoa1 (g/L) | *GYS2* | glycogen synthase 2 (liver) | 0.8 |
| TRLapob (mg/L) | *ALG3* | asparagine-linked glycosylation 3, alpha-1,3- mannosyltransferase homolog (S. cerevisiae) | -0.8 |
| TRLapob (mg/L) | *A4GALT* | alpha 1,4-galactosyltransferase | -0.75 |
| TRLapob (mg/L) | *GNPNAT1* | glucosamine-phosphate N-acetyltransferase 1 | 0.8 |
| TRLapob (mg/L) | *GGPS1* | geranylgeranyl diphosphate synthase 1 | 0.76 |
| C14:0 (mg/mL) | *LTC4S* | leukotriene C4 synthase | 0.75 |
| C18:0 (mg/mL) | *CDO1* | cysteine dioxygenase, type I | 0.79 |
| C18:0 (mg/mL) | *SPTLC1* | serine palmitoyltransferase, long chain base subunit 1 | 0.8 |
| C18:0 (mg/mL) | *CEL* | carboxyl ester lipase (bile salt-stimulated lipase) | -0.84 |
| C18:0 (mg/mL) | *FARS2* | phenylalanyl-tRNA synthetase 2, mitochondrial | 0.78 |
| C18:0 (mg/mL) | *PTGES3* | prostaglandin E synthase 3 (cytosolic) | 0.77 |
| C18:0 (mg/mL) | *MTFMT* | mitochondrial methionyl-tRNA formyltransferase | 0.76 |
| C18:0 (mg/mL) | *ADH5* | alcohol dehydrogenase 5 (class III), chi polypeptide | 0.81 |
| C18:0 (mg/mL) | *DDOST* | dolichyl-diphosphooligosaccharide--protein glycosyltransferase | 0.78 |
| C18:0 (mg/mL) | *DUT* | deoxyuridine triphosphatase | 0.77 |
| C18:0 (mg/mL) | *ACSL1* | acyl-CoA synthetase long-chain family member 1 | 0.76 |
| C18:0 (mg/mL) | *GAA* | glucosidase, alpha; acid | -0.79 |
| C18:0 (mg/mL) | *HADHB* | hydroxyacyl-CoA dehydrogenase/3-ketoacyl-CoA thiolase/enoyl-CoA hydratase (trifunctional protein), beta subunit | 0.8 |
| C18:0 (mg/mL) | *HSD17B4* | hydroxysteroid (17-beta) dehydrogenase 4 | 0.75 |
| C18:0 (mg/mL) | *GSTK1* | glutathione S-transferase kappa 1 | 0.76 |
| C18:0 (mg/mL) | *LDHB* | lactate dehydrogenase B | 0.75 |
| C18:0 (mg/mL) | *MAOA* | monoamine oxidase A | 0.78 |
| C18:0 (mg/mL) | *MGST1* | microsomal glutathione S-transferase 1 | 0.75 |
| C18:0 (mg/mL) | *MGST3* | microsomal glutathione S-transferase 3 | 0.77 |
| C18:0 (mg/mL) | *NDUFB3* | NADH dehydrogenase (ubiquinone) 1 beta subcomplex, 3, 12kDa | 0.76 |
| C18:0 (mg/mL) | *NDUFB9* | NADH dehydrogenase (ubiquinone) 1 beta subcomplex, 9, 22kDa | 0.76 |
| C18:0 (mg/mL) | *NDUFS4* | NADH dehydrogenase (ubiquinone) Fe-S protein 4, 18kDa (NADH-coenzyme Q reductase) | 0.76 |
| C18:0 (mg/mL) | *NEU1* | sialidase 1 (lysosomal sialidase) | 0.77 |
| C18:0 (mg/mL) | *ATP2B4* | ATPase, Ca++ transporting, plasma membrane 4 | 0.77 |
| C18:0 (mg/mL) | *AK3* | adenylate kinase 3 | 0.75 |
| C18:0 (mg/mL) | *NSDHL* | NAD(P) dependent steroid dehydrogenase-like | 0.76 |
| C18:0 (mg/mL) | *ATP5C1* | ATP synthase, H+ transporting, mitochondrial F1 complex, gamma polypeptide 1 | 0.78 |
| C18:0 (mg/mL) | *ATP6V1D* | ATPase, H+ transporting, lysosomal 34kDa, V1 subunit D | 0.78 |
| C18:0 (mg/mL) | *ATP5E* | ATP synthase, H+ transporting, mitochondrial F1 complex, epsilon subunit | 0.79 |
| C18:0 (mg/mL) | *ATP5G2* | ATP synthase, H+ transporting, mitochondrial Fo complex, subunit C2 (subunit 9) | 0.76 |
| C18:0 (mg/mL) | *CMPK1* | cytidine monophosphate (UMP-CMP) kinase 1, cytosolic | 0.82 |
| C18:0 (mg/mL) | *ATP5G3* | ATP synthase, H+ transporting, mitochondrial Fo complex, subunit C3 (subunit 9) | 0.77 |
| C18:0 (mg/mL) | *ACP1* | acid phosphatase 1, soluble | 0.8 |
| C18:0 (mg/mL) | *ATP5J* | ATP synthase, H+ transporting, mitochondrial Fo complex, subunit F6 | 0.76 |
| C18:0 (mg/mL) | *PIGF* | phosphatidylinositol glycan anchor biosynthesis, class F | 0.79 |
| C18:0 (mg/mL) | *PLA2G5* | phospholipase A2, group V | -0.79 |
| C18:0 (mg/mL) | *NUDT9* | nudix (nucleoside diphosphate linked moiety X)-type motif 9 | 0.84 |
| C18:0 (mg/mL) | *POLB* | polymerase (DNA directed), beta | 0.76 |
| C18:0 (mg/mL) | *OXSM* | 3-oxoacyl-ACP synthase, mitochondrial | 0.81 |
| C18:0 (mg/mL) | *CSGALNACT1* | chondroitin sulfate N-acetylgalactosaminyltransferase 1 | 0.77 |
| C18:0 (mg/mL) | *NDUFA12* | NADH dehydrogenase (ubiquinone) 1 alpha subcomplex, 12 | 0.76 |
| C18:0 (mg/mL) | *CHPT1* | choline phosphotransferase 1 | 0.83 |
| C18:0 (mg/mL) | *PSPH* | phosphoserine phosphatase | 0.77 |
| C18:0 (mg/mL) | *GPAM* | glycerol-3-phosphate acyltransferase, mitochondrial | 0.79 |
| C18:0 (mg/mL) | *SAT1* | spermidine/spermine N1-acetyltransferase 1 | 0.8 |
| C18:0 (mg/mL) | *SDHB* | succinate dehydrogenase complex, subunit B, iron sulfur (Ip) | 0.75 |
| C18:0 (mg/mL) | *SDHD* | succinate dehydrogenase complex, subunit D, integral membrane protein | 0.77 |
| C18:0 (mg/mL) | *NADK* | NAD kinase | 0.78 |
| C18:0 (mg/mL) | *BPGM* | 2,3-bisphosphoglycerate mutase | 0.75 |
| C18:0 (mg/mL) | *TALDO1* | transaldolase 1 | 0.78 |
| C18:0 (mg/mL) | *UGP2* | UDP-glucose pyrophosphorylase 2 | 0.76 |
| C18:0 (mg/mL) | *PPCS* | phosphopantothenoylcysteine synthetase | 0.79 |
| C18:0 (mg/mL) | *PIP5K1A* | phosphatidylinositol-4-phosphate 5-kinase, type I, alpha | -0.78 |
| C18:0 (mg/mL) | *POLR3GL* | polymerase (RNA) III (DNA directed) polypeptide G (32kD)-like | 0.8 |
| C18:0 (mg/mL) | *MCEE* | methylmalonyl CoA epimerase | 0.76 |
| C18:0 (mg/mL) | *AGPS* | alkylglycerone phosphate synthase | 0.76 |
| C18:0 (mg/mL) | *SUCLA2* | succinate-CoA ligase, ADP-forming, beta subunit | 0.78 |
| C18:0 (mg/mL) | *PLCZ1* | phospholipase C, zeta 1 | -0.81 |
| C18:0 (mg/mL) | *GSTO1* | glutathione S-transferase omega 1 | 0.76 |
| C18:0 (mg/mL) | *ENTPD6* | ectonucleoside triphosphate diphosphohydrolase 6 (putative) | -0.79 |
| C18:0 (mg/mL) | *ATP6V1G1* | ATPase, H+ transporting, lysosomal 13kDa, V1 subunit G1 | 0.78 |
| C18:2 (n-6) (mg/mL) | *ACSL1* | acyl-CoA synthetase long-chain family member 1 | 0.76 |
| C18:2 (n-6) (mg/mL) | *ATP6V1D* | ATPase, H+ transporting, lysosomal 34kDa, V1 subunit D | 0.77 |
| C18:2 (n-6) (mg/mL) | *PDHX* | pyruvate dehydrogenase complex, component X | 0.76 |
| C18:2 (n-6) (mg/mL) | *DPM1* | dolichyl-phosphate mannosyltransferase polypeptide 1, catalytic subunit | 0.76 |
| C20:1 (mg/mL) | *ADSS* | adenylosuccinate synthase | -0.82 |
| C20:1 (mg/mL) | *HMGCR* | 3-hydroxy-3-methylglutaryl-CoA reductase | -0.76 |
| C20:1 (mg/mL) | *MGAT2* | mannosyl (alpha-1,6-)-glycoprotein beta-1,2-N-acetylglucosaminyltransferase | -0.79 |
| C20:1 (mg/mL) | *ATP6V1G2* | ATPase, H+ transporting, lysosomal 13kDa, V1 subunit G2 | 0.8 |
| C20:1 (mg/mL) | *DDA1* | DET1 and DDB1 associated 1 | 0.84 |
| C20:1 (mg/mL) | *KATNAL1* | katanin p60 subunit A-like 1 | -0.78 |
| C20:3 (n-6) (mg/mL) | *CDO1* | cysteine dioxygenase, type I | 0.75 |
| C20:3 (n-6) (mg/mL) | *SPTLC1* | serine palmitoyltransferase, long chain base subunit 1 | 0.75 |
| C20:3 (n-6) (mg/mL) | *CEL* | carboxyl ester lipase (bile salt-stimulated lipase) | -0.8 |
| C20:3 (n-6) (mg/mL) | *ADH5* | alcohol dehydrogenase 5 (class III), chi polypeptide | 0.79 |
| C20:3 (n-6) (mg/mL) | *DDOST* | dolichyl-diphosphooligosaccharide--protein glycosyltransferase | 0.78 |
| C20:3 (n-6) (mg/mL) | *ALDH3A2* | aldehyde dehydrogenase 3 family, member A2 | 0.76 |
| C20:3 (n-6) (mg/mL) | *MAT2B* | methionine adenosyltransferase II, beta | 0.75 |
| C20:3 (n-6) (mg/mL) | *CMPK1* | cytidine monophosphate (UMP-CMP) kinase 1, cytosolic | 0.77 |
| C20:3 (n-6) (mg/mL) | *ACP1* | acid phosphatase 1, soluble | 0.76 |
| C20:3 (n-6) (mg/mL) | *PIGF* | phosphatidylinositol glycan anchor biosynthesis, class F | 0.76 |
| C20:3 (n-6) (mg/mL) | *PIGH* | phosphatidylinositol glycan anchor biosynthesis, class H | 0.75 |
| C20:3 (n-6) (mg/mL) | *NUDT9* | nudix (nucleoside diphosphate linked moiety X)-type motif 9 | 0.8 |
| C20:3 (n-6) (mg/mL) | *CSGALNACT1* | chondroitin sulfate N-acetylgalactosaminyltransferase 1 | 0.76 |
| C20:3 (n-6) (mg/mL) | *CHPT1* | choline phosphotransferase 1 | 0.81 |
| C20:3 (n-6) (mg/mL) | *PSPH* | phosphoserine phosphatase | 0.77 |
| C20:3 (n-6) (mg/mL) | *NADK* | NAD kinase | 0.78 |
| C20:3 (n-6) (mg/mL) | *PIP5K1A* | phosphatidylinositol-4-phosphate 5-kinase, type I, alpha | -0.76 |
| C20:4 (n-6) (mg/mL) | *MAT2B* | methionine adenosyltransferase II, beta | 0.78 |
| C20:4 (n-6) (mg/mL) | *ARSD* | arylsulfatase D | -0.79 |
| C20:4 (n-6) (mg/mL) | *PIP4K2A* | phosphatidylinositol-5-phosphate 4-kinase, type II, alpha | 0.76 |
| C20:4 (n-3) (mg/mL) | *ALG3* | asparagine-linked glycosylation 3, alpha-1,3- mannosyltransferase homolog (S. cerevisiae) | -0.86 |
| C20:5 (n-3) (mg/mL) | *ATP9A* | ATPase, class II, type 9A | 0.76 |
| C20:5 (n-3) (mg/mL) | *CDIPT* | CDP-diacylglycerol--inositol 3-phosphatidyltransferase | 0.78 |
| C20:5 (n-3) (mg/mL) | *PMVK* | phosphomevalonate kinase | 0.78 |
| C20:5 (n-3) (mg/mL) | *ADH5* | alcohol dehydrogenase 5 (class III), chi polypeptide | 0.76 |
| C20:5 (n-3) (mg/mL) | *CYP4B1* | cytochrome P450, family 4, subfamily B, polypeptide 1 | 0.78 |
| C20:5 (n-3) (mg/mL) | *EEF2* | eukaryotic translation elongation factor 2 | 0.75 |
| C20:5 (n-3) (mg/mL) | *ALDH2* | aldehyde dehydrogenase 2 family (mitochondrial) | 0.79 |
| C20:5 (n-3) (mg/mL) | *FDFT1* | farnesyl-diphosphate farnesyltransferase 1 | 0.77 |
| C20:5 (n-3) (mg/mL) | *MLYCD* | malonyl-CoA decarboxylase | 0.76 |
| C20:5 (n-3) (mg/mL) | *GALT* | galactose-1-phosphate uridylyltransferase | 0.79 |
| C20:5 (n-3) (mg/mL) | *GCK* | glucokinase (hexokinase 4) | -0.84 |
| C20:5 (n-3) (mg/mL) | *GPD1* | glycerol-3-phosphate dehydrogenase 1 (soluble) | 0.78 |
| C20:5 (n-3) (mg/mL) | *GSTP1* | glutathione S-transferase pi 1 | 0.75 |
| C20:5 (n-3) (mg/mL) | *APOBEC4* | apolipoprotein B mRNA editing enzyme, catalytic polypeptide-like 4 (putative) | -0.81 |
| C20:5 (n-3) (mg/mL) | *MAOA* | monoamine oxidase A | 0.77 |
| C20:5 (n-3) (mg/mL) | *A4GALT* | alpha 1,4-galactosyltransferase | -0.79 |
| C20:5 (n-3) (mg/mL) | *MLL5* | myeloid/lymphoid or mixed-lineage leukemia 5 (trithorax homolog, Drosophila) | -0.79 |
| C20:5 (n-3) (mg/mL) | *PRPS1* | phosphoribosyl pyrophosphate synthetase 1 | 0.75 |
| C20:5 (n-3) (mg/mL) | *WHSC1* | Wolf-Hirschhorn syndrome candidate 1 | 0.76 |
| C20:5 (n-3) (mg/mL) | *AOC3* | amine oxidase, copper containing 3 (vascular adhesion protein 1) | 0.76 |
| C20:5 (n-3) (mg/mL) | *GGPS1* | geranylgeranyl diphosphate synthase 1 | 0.85 |
| C22:5 (n-3) (mg/mL) | *GSS* | glutathione synthetase | -0.82 |
| C22:5 (n-3) (mg/mL) | *HMOX1* | heme oxygenase (decycling) 1 | -0.76 |
| C22:6 (n-3) (mg/mL) | *CEPT1* | choline/ethanolamine phosphotransferase 1 | 0.77 |
| C22:6 (n-3) (mg/mL) | *SPTLC1* | serine palmitoyltransferase, long chain base subunit 1 | 0.77 |
| C22:6 (n-3) (mg/mL) | *CEL* | carboxyl ester lipase (bile salt-stimulated lipase) | -0.8 |
| C22:6 (n-3) (mg/mL) | *POLQ* | polymerase (DNA directed), theta | -0.75 |
| C22:6 (n-3) (mg/mL) | *MGEA5* | meningioma expressed antigen 5 (hyaluronidase) | 0.84 |
| C22:6 (n-3) (mg/mL) | *PTGES3* | prostaglandin E synthase 3 (cytosolic) | 0.78 |
| C22:6 (n-3) (mg/mL) | *PAPOLA* | poly(A) polymerase alpha | 0.78 |
| C22:6 (n-3) (mg/mL) | *POLS* | PAP-associated domain-containing protein 7 | 0.77 |
| C22:6 (n-3) (mg/mL) | *POLI* | polymerase (DNA directed) iota | 0.76 |
| C22:6 (n-3) (mg/mL) | *SAT2* | spermidine/spermine N1-acetyltransferase family member 2 | 0.8 |
| C22:6 (n-3) (mg/mL) | *GALNT13* | UDP-N-acetyl-alpha-D-galactosamine:polypeptide N-acetylgalactosaminyltransferase 13 (GalNAc-T13) | -0.8 |
| C22:6 (n-3) (mg/mL) | *MTFMT* | mitochondrial methionyl-tRNA formyltransferase | 0.76 |
| C22:6 (n-3) (mg/mL) | *ADH5* | alcohol dehydrogenase 5 (class III), chi polypeptide | 0.8 |
| C22:6 (n-3) (mg/mL) | *DGKA* | diacylglycerol kinase, alpha 80kDa | 0.83 |
| C22:6 (n-3) (mg/mL) | *DLD* | dihydrolipoamide dehydrogenase | 0.8 |
| C22:6 (n-3) (mg/mL) | *DPYD* | dihydropyrimidine dehydrogenase | 0.77 |
| C22:6 (n-3) (mg/mL) | *EEF1A1* | eukaryotic translation elongation factor 1 alpha 1 | 0.77 |
| C22:6 (n-3) (mg/mL) | *EEF2* | eukaryotic translation elongation factor 2 | 0.77 |
| C22:6 (n-3) (mg/mL) | *PIKFYVE* | phosphoinositide kinase, FYVE finger containing | 0.8 |
| C22:6 (n-3) (mg/mL) | *ALDH2* | aldehyde dehydrogenase 2 family (mitochondrial) | 0.79 |
| C22:6 (n-3) (mg/mL) | *FDFT1* | farnesyl-diphosphate farnesyltransferase 1 | 0.76 |
| C22:6 (n-3) (mg/mL) | *FH* | fumarate hydratase | 0.79 |
| C22:6 (n-3) (mg/mL) | *PTGR1* | prostaglandin reductase 1 | 0.75 |
| C22:6 (n-3) (mg/mL) | *PLCL2* | phospholipase C-like 2 | 0.83 |
| C22:6 (n-3) (mg/mL) | *HARS2* | histidyl-tRNA synthetase 2, mitochondrial (putative) | 0.78 |
| C22:6 (n-3) (mg/mL) | *FUT3* | fucosyltransferase 3 (galactoside 3(4)-L-fucosyltransferase, Lewis blood group) | -0.83 |
| C22:6 (n-3) (mg/mL) | *GAA* | glucosidase, alpha; acid | -0.82 |
| C22:6 (n-3) (mg/mL) | *GLUD1* | glutamate dehydrogenase 1 | 0.79 |
| C22:6 (n-3) (mg/mL) | *SETD2* | SET domain containing 2 | 0.79 |
| C22:6 (n-3) (mg/mL) | *HADHB* | hydroxyacyl-CoA dehydrogenase/3-ketoacyl-CoA thiolase/enoyl-CoA hydratase (trifunctional protein), beta subunit | 0.83 |
| C22:6 (n-3) (mg/mL) | *HEXB* | hexosaminidase B (beta polypeptide) | 0.8 |
| C22:6 (n-3) (mg/mL) | *HSD17B4* | hydroxysteroid (17-beta) dehydrogenase 4 | 0.79 |
| C22:6 (n-3) (mg/mL) | *POLN* | polymerase (DNA directed) nu | -0.76 |
| C22:6 (n-3) (mg/mL) | *KARS* | lysyl-tRNA synthetase | 0.85 |
| C22:6 (n-3) (mg/mL) | *ACAT1* | acetyl-CoA acetyltransferase 1 | 0.78 |
| C22:6 (n-3) (mg/mL) | *LDHB* | lactate dehydrogenase B | 0.76 |
| C22:6 (n-3) (mg/mL) | *LPL* | lipoprotein lipase | 0.78 |
| C22:6 (n-3) (mg/mL) | *LTA4H* | leukotriene A4 hydrolase | 0.79 |
| C22:6 (n-3) (mg/mL) | *MGST1* | microsomal glutathione S-transferase 1 | 0.78 |
| C22:6 (n-3) (mg/mL) | *MGST3* | microsomal glutathione S-transferase 3 | 0.78 |
| C22:6 (n-3) (mg/mL) | *ASAH1* | N-acylsphingosine amidohydrolase (acid ceramidase) 1 | 0.77 |
| C22:6 (n-3) (mg/mL) | *NARS* | asparaginyl-tRNA synthetase | 0.78 |
| C22:6 (n-3) (mg/mL) | *NDUFA2* | NADH dehydrogenase (ubiquinone) 1 alpha subcomplex, 2, 8kDa | 0.79 |
| C22:6 (n-3) (mg/mL) | *NDUFA6* | NADH dehydrogenase (ubiquinone) 1 alpha subcomplex, 6, 14kDa | 0.76 |
| C22:6 (n-3) (mg/mL) | *NDUFB1* | NADH dehydrogenase (ubiquinone) 1 beta subcomplex, 1, 7kDa | 0.78 |
| C22:6 (n-3) (mg/mL) | *NDUFB2* | NADH dehydrogenase (ubiquinone) 1 beta subcomplex, 2, 8kDa | 0.75 |
| C22:6 (n-3) (mg/mL) | *NDUFB3* | NADH dehydrogenase (ubiquinone) 1 beta subcomplex, 3, 12kDa | 0.78 |
| C22:6 (n-3) (mg/mL) | *NDUFB5* | NADH dehydrogenase (ubiquinone) 1 beta subcomplex, 5, 16kDa | 0.78 |
| C22:6 (n-3) (mg/mL) | *NDUFB8* | NADH dehydrogenase (ubiquinone) 1 beta subcomplex, 8, 19kDa | 0.78 |
| C22:6 (n-3) (mg/mL) | *NDUFS4* | NADH dehydrogenase (ubiquinone) Fe-S protein 4, 18kDa (NADH-coenzyme Q reductase) | 0.76 |
| C22:6 (n-3) (mg/mL) | *NEU1* | sialidase 1 (lysosomal sialidase) | 0.77 |
| C22:6 (n-3) (mg/mL) | *ATP2B4* | ATPase, Ca++ transporting, plasma membrane 4 | 0.78 |
| C22:6 (n-3) (mg/mL) | *OAT* | ornithine aminotransferase | 0.85 |
| C22:6 (n-3) (mg/mL) | *ATP5A1* | ATP synthase, H+ transporting, mitochondrial F1 complex, alpha subunit 1, cardiac muscle | 0.82 |
| C22:6 (n-3) (mg/mL) | *PAFAH1B1* | platelet-activating factor acetylhydrolase 1b, regulatory subunit 1 (45kDa) | 0.8 |
| C22:6 (n-3) (mg/mL) | *ATP5B* | ATP synthase, H+ transporting, mitochondrial F1 complex, beta polypeptide | 0.8 |
| C22:6 (n-3) (mg/mL) | *AK3* | adenylate kinase 3 | 0.81 |
| C22:6 (n-3) (mg/mL) | *ATP5C1* | ATP synthase, H+ transporting, mitochondrial F1 complex, gamma polypeptide 1 | 0.77 |
| C22:6 (n-3) (mg/mL) | *LAP3* | leucine aminopeptidase 3 | 0.82 |
| C22:6 (n-3) (mg/mL) | *POLR1D* | polymerase (RNA) I polypeptide D, 16kDa | 0.78 |
| C22:6 (n-3) (mg/mL) | *GMPR2* | guanosine monophosphate reductase 2 | 0.82 |
| C22:6 (n-3) (mg/mL) | *ATP5G2* | ATP synthase, H+ transporting, mitochondrial Fo complex, subunit C2 (subunit 9) | 0.81 |
| C22:6 (n-3) (mg/mL) | *CMPK1* | cytidine monophosphate (UMP-CMP) kinase 1, cytosolic | 0.79 |
| C22:6 (n-3) (mg/mL) | *ATP5G3* | ATP synthase, H+ transporting, mitochondrial Fo complex, subunit C3 (subunit 9) | 0.76 |
| C22:6 (n-3) (mg/mL) | *ATP5J* | ATP synthase, H+ transporting, mitochondrial Fo complex, subunit F6 | 0.8 |
| C22:6 (n-3) (mg/mL) | *PGK1* | phosphoglycerate kinase 1 | 0.79 |
| C22:6 (n-3) (mg/mL) | *PGK2* | phosphoglycerate kinase 2 | -0.77 |
| C22:6 (n-3) (mg/mL) | *PHYH* | phytanoyl-CoA 2-hydroxylase | 0.79 |
| C22:6 (n-3) (mg/mL) | *PIK3C3* | phosphoinositide-3-kinase, class 3 | 0.81 |
| C22:6 (n-3) (mg/mL) | *ATP6V1E1* | ATPase, H+ transporting, lysosomal 31kDa, V1 subunit E1 | 0.83 |
| C22:6 (n-3) (mg/mL) | *PIK3CA* | phosphoinositide-3-kinase, catalytic, alpha polypeptide | 0.77 |
| C22:6 (n-3) (mg/mL) | *ATP5O* | ATP synthase, H+ transporting, mitochondrial F1 complex, O subunit | 0.82 |
| C22:6 (n-3) (mg/mL) | *POLB* | polymerase (DNA directed), beta | 0.79 |
| C22:6 (n-3) (mg/mL) | *POLR2C* | polymerase (RNA) II (DNA directed) polypeptide C, 33kDa | 0.76 |
| C22:6 (n-3) (mg/mL) | *SMOX* | spermine oxidase | -0.76 |
| C22:6 (n-3) (mg/mL) | *KIAA1310* | KIAA1310 | 0.78 |
| C22:6 (n-3) (mg/mL) | *IARS2* | isoleucyl-tRNA synthetase 2, mitochondrial | 0.79 |
| C22:6 (n-3) (mg/mL) | *PECR* | peroxisomal trans-2-enoyl-CoA reductase | 0.76 |
| C22:6 (n-3) (mg/mL) | *CMAS* | cytidine monophosphate N-acetylneuraminic acid synthetase | 0.84 |
| C22:6 (n-3) (mg/mL) | *NDUFA12* | NADH dehydrogenase (ubiquinone) 1 alpha subcomplex, 12 | 0.77 |
| C22:6 (n-3) (mg/mL) | *CTPS2* | CTP synthase II | 0.81 |
| C22:6 (n-3) (mg/mL) | *GPAM* | glycerol-3-phosphate acyltransferase, mitochondrial | 0.79 |
| C22:6 (n-3) (mg/mL) | *PYGL* | phosphorylase, glycogen, liver | 0.77 |
| C22:6 (n-3) (mg/mL) | *MLL3* | myeloid/lymphoid or mixed-lineage leukemia 3 | 0.76 |
| C22:6 (n-3) (mg/mL) | *BCKDHB* | branched chain keto acid dehydrogenase E1, beta polypeptide | 0.77 |
| C22:6 (n-3) (mg/mL) | *SAT1* | spermidine/spermine N1-acetyltransferase 1 | 0.81 |
| C22:6 (n-3) (mg/mL) | *SCP2* | sterol carrier protein 2 | 0.83 |
| C22:6 (n-3) (mg/mL) | *SDHB* | succinate dehydrogenase complex, subunit B, iron sulfur (Ip) | 0.83 |
| C22:6 (n-3) (mg/mL) | *POLR1E* | polymerase (RNA) I polypeptide E, 53kDa | 0.79 |
| C22:6 (n-3) (mg/mL) | *BLVRB* | biliverdin reductase B (flavin reductase (NADPH)) | 0.84 |
| C22:6 (n-3) (mg/mL) | *CERK* | ceramide kinase | 0.76 |
| C22:6 (n-3) (mg/mL) | *CYP4F12* | cytochrome P450, family 4, subfamily F, polypeptide 12 | -0.82 |
| C22:6 (n-3) (mg/mL) | *TAF9* | TAF9 RNA polymerase II, TATA box binding protein (TBP)-associated factor, 32kDa | 0.77 |
| C22:6 (n-3) (mg/mL) | *TALDO1* | transaldolase 1 | 0.8 |
| C22:6 (n-3) (mg/mL) | *UGP2* | UDP-glucose pyrophosphorylase 2 | 0.78 |
| C22:6 (n-3) (mg/mL) | *UROD* | uroporphyrinogen decarboxylase | 0.79 |
| C22:6 (n-3) (mg/mL) | *SAP130* | Sin3A-associated protein, 130kDa | 0.79 |
| C22:6 (n-3) (mg/mL) | *PPCS* | phosphopantothenoylcysteine synthetase | 0.83 |
| C22:6 (n-3) (mg/mL) | *UXS1* | UDP-glucuronate decarboxylase 1 | 0.78 |
| C22:6 (n-3) (mg/mL) | *TKTL2* | transketolase-like 2 | -0.78 |
| C22:6 (n-3) (mg/mL) | *POLR3GL* | polymerase (RNA) III (DNA directed) polypeptide G (32kD)-like | 0.8 |
| C22:6 (n-3) (mg/mL) | *GNPAT* | glyceronephosphate O-acyltransferase | 0.85 |
| C22:6 (n-3) (mg/mL) | *MCEE* | methylmalonyl CoA epimerase | 0.78 |
| C22:6 (n-3) (mg/mL) | *AGPS* | alkylglycerone phosphate synthase | 0.77 |
| C22:6 (n-3) (mg/mL) | *CDS2* | CDP-diacylglycerol synthase (phosphatidate cytidylyltransferase) 2 | 0.77 |
| C22:6 (n-3) (mg/mL) | *SUCLG2* | succinate-CoA ligase, GDP-forming, beta subunit | 0.75 |
| C22:6 (n-3) (mg/mL) | *SUCLG1* | succinate-CoA ligase, alpha subunit | 0.84 |
| C22:6 (n-3) (mg/mL) | *SUCLA2* | succinate-CoA ligase, ADP-forming, beta subunit | 0.78 |
| C22:6 (n-3) (mg/mL) | *ATP6V0E1* | ATPase, H+ transporting, lysosomal 9kDa, V0 subunit e1 | 0.79 |
| C22:6 (n-3) (mg/mL) | *PAPSS1* | 3'-phosphoadenosine 5'-phosphosulfate synthase 1 | 0.78 |
| C22:6 (n-3) (mg/mL) | *ENTPD6* | ectonucleoside triphosphate diphosphohydrolase 6 (putative) | -0.83 |
| C22:6 (n-3) (mg/mL) | *ATP6V1G1* | ATPase, H+ transporting, lysosomal 13kDa, V1 subunit G1 | 0.81 |
| C22:6 (n-3) (mg/mL) | *PRDX6* | peroxiredoxin 6 | 0.81 |
| TRLC (mmol/L) | *ALG3* | asparagine-linked glycosylation 3, alpha-1,3- mannosyltransferase homolog (S. cerevisiae) | -0.75 |
| TRLC (mmol/L) | *ATP6V1G3* | ATPase, H+ transporting, lysosomal 13kDa, V1 subunit G3 | -0.8 |
| THDL (mmol/L) | *GYS2* | glycogen synthase 2 (liver) | 0.76 |
