# Supplementary material for: Transcriptomic Coordination in the Human Metabolic Network Reveals Links between n-3 Fat Intake, Adipose Tissue Gene Expression and Metabolic Health
Source: PLoS Comput Biol. 2011 Nov 3;7(11):e1002223. doi: 10.1371/journal.pcbi.1002223 (PMC3207936; doi:10.1371/journal.pcbi.1002223)
Supplement: Table S5 — Results from rCCA of adipose tissue gene expression and plasma cytokines, IVGTT measurements, prostaglandin and urinary isoprostane. Plasma marker-gene pairs passing the similarity threshold of 0.7 are shown. (DOCX) [file pcbi.1002223.s007.docx]

**Supplementary Table S5.** Results from rCCA of adipose tissue gene expression and plasma cytokines, IVGTT measurements, prostaglandin and urinary isoprostane. Plasma marker-gene pairs passing the similarity threshold of 0.7 are shown.

| **Plasma marker** | **Gene** | **Gene name** | **Similarity score** |
| --- | --- | --- | --- |
| cpeptide (ng/ml) | *PAFAH1B2* | platelet-activating factor acetylhydrolase 1b, catalytic subunit 2 (30kDa) | 0.71 |
| cpeptide (ng/ml) | *PFKFB4* | 6-phosphofructo-2-kinase/fructose-2,6-biphosphatase 4 | 0.71 |
| crp (mg/ml) | *CSAD* | cysteine sulfinic acid decarboxylase | -0.74 |
| 15-keto-PGF2a (mmol/mmol creatinine) | *AMPD3* | adenosine monophosphate deaminase 3 | -0.7 |
| 15-keto-PGF2a (mmol/mmol creatinine) | *PCCB* | propionyl CoA carboxylase, beta polypeptide | -0.76 |
| 8-iso-PGF2a (mmol/mmol creatinine) | *NME6* | non-metastatic cells 6, protein expressed in (nucleoside-diphosphate kinase) | -0.75 |
| 8-iso-PGF2a (mmol/mmol creatinine) | *CDO1* | cysteine dioxygenase, type I | -0.74 |
| 8-iso-PGF2a (mmol/mmol creatinine) | *SPTLC1* | serine palmitoyltransferase, long chain base subunit 1 | -0.76 |
| 8-iso-PGF2a (mmol/mmol creatinine) | *CEL* | carboxyl ester lipase (bile salt-stimulated lipase) | 0.72 |
| 8-iso-PGF2a (mmol/mmol creatinine) | *FARS2* | phenylalanyl-tRNA synthetase 2, mitochondrial | -0.78 |
| 8-iso-PGF2a (mmol/mmol creatinine) | *PTGES3* | prostaglandin E synthase 3 (cytosolic) | -0.73 |
| 8-iso-PGF2a (mmol/mmol creatinine) | *PAPOLA* | poly(A) polymerase alpha | -0.74 |
| 8-iso-PGF2a (mmol/mmol creatinine) | *POLI* | polymerase (DNA directed) iota | -0.72 |
| 8-iso-PGF2a (mmol/mmol creatinine) | *CPOX* | coproporphyrinogen oxidase | -0.75 |
| 8-iso-PGF2a (mmol/mmol creatinine) | *DPYD* | dihydropyrimidine dehydrogenase | -0.77 |
| 8-iso-PGF2a (mmol/mmol creatinine) | *EEF2* | eukaryotic translation elongation factor 2 | -0.7 |
| 8-iso-PGF2a (mmol/mmol creatinine) | *ACSL1* | acyl-CoA synthetase long-chain family member 1 | -0.73 |
| 8-iso-PGF2a (mmol/mmol creatinine) | *FDFT1* | farnesyl-diphosphate farnesyltransferase 1 | -0.71 |
| 8-iso-PGF2a (mmol/mmol creatinine) | *FH* | fumarate hydratase | -0.73 |
| 8-iso-PGF2a (mmol/mmol creatinine) | *ALOX5* | arachidonate 5-lipoxygenase | -0.71 |
| 8-iso-PGF2a (mmol/mmol creatinine) | *GPX6* | glutathione peroxidase 6 (olfactory) | 0.72 |
| 8-iso-PGF2a (mmol/mmol creatinine) | *SGMS1* | sphingomyelin synthase 1 | -0.73 |
| 8-iso-PGF2a (mmol/mmol creatinine) | *GLA* | galactosidase, alpha | -0.77 |
| 8-iso-PGF2a (mmol/mmol creatinine) | *GUK1* | guanylate kinase 1 | -0.76 |
| 8-iso-PGF2a (mmol/mmol creatinine) | *ALG5* | asparagine-linked glycosylation 5, dolichyl-phosphate beta-glucosyltransferase homolog (S. cerevisiae) | -0.71 |
| 8-iso-PGF2a (mmol/mmol creatinine) | *HADHB* | hydroxyacyl-CoA dehydrogenase/3-ketoacyl-CoA thiolase/enoyl-CoA hydratase (trifunctional protein), beta subunit | -0.7 |
| 8-iso-PGF2a (mmol/mmol creatinine) | *HMGCL* | 3-hydroxymethyl-3-methylglutaryl-CoA lyase | -0.77 |
| 8-iso-PGF2a (mmol/mmol creatinine) | *ACADL* | acyl-CoA dehydrogenase, long chain | -0.7 |
| 8-iso-PGF2a (mmol/mmol creatinine) | *POLN* | polymerase (DNA directed) nu | 0.74 |
| 8-iso-PGF2a (mmol/mmol creatinine) | *IMPA1* | inositol(myo)-1(or 4)-monophosphatase 1 | -0.7 |
| 8-iso-PGF2a (mmol/mmol creatinine) | *GSTK1* | glutathione S-transferase kappa 1 | -0.73 |
| 8-iso-PGF2a (mmol/mmol creatinine) | *KLK1* | kallikrein 1 | 0.74 |
| 8-iso-PGF2a (mmol/mmol creatinine) | *LDHB* | lactate dehydrogenase B | -0.75 |
| 8-iso-PGF2a (mmol/mmol creatinine) | *LTA4H* | leukotriene A4 hydrolase | -0.75 |
| 8-iso-PGF2a (mmol/mmol creatinine) | *MGST1* | microsomal glutathione S-transferase 1 | -0.73 |
| 8-iso-PGF2a (mmol/mmol creatinine) | *MGST3* | microsomal glutathione S-transferase 3 | -0.72 |
| 8-iso-PGF2a (mmol/mmol creatinine) | *NDUFA2* | NADH dehydrogenase (ubiquinone) 1 alpha subcomplex, 2, 8kDa | -0.74 |
| 8-iso-PGF2a (mmol/mmol creatinine) | *NDUFA3* | NADH dehydrogenase (ubiquinone) 1 alpha subcomplex, 3, 9kDa | -0.71 |
| 8-iso-PGF2a (mmol/mmol creatinine) | *NDUFB1* | NADH dehydrogenase (ubiquinone) 1 beta subcomplex, 1, 7kDa | -0.72 |
| 8-iso-PGF2a (mmol/mmol creatinine) | *NDUFB2* | NADH dehydrogenase (ubiquinone) 1 beta subcomplex, 2, 8kDa | -0.72 |
| 8-iso-PGF2a (mmol/mmol creatinine) | *NDUFB3* | NADH dehydrogenase (ubiquinone) 1 beta subcomplex, 3, 12kDa | -0.75 |
| 8-iso-PGF2a (mmol/mmol creatinine) | *NDUFB4* | NADH dehydrogenase (ubiquinone) 1 beta subcomplex, 4, 15kDa | -0.8 |
| 8-iso-PGF2a (mmol/mmol creatinine) | *NDUFB5* | NADH dehydrogenase (ubiquinone) 1 beta subcomplex, 5, 16kDa | -0.74 |
| 8-iso-PGF2a (mmol/mmol creatinine) | *NDUFC1* | NADH dehydrogenase (ubiquinone) 1, subcomplex unknown, 1, 6kDa | -0.72 |
| 8-iso-PGF2a (mmol/mmol creatinine) | *NDUFS4* | NADH dehydrogenase (ubiquinone) Fe-S protein 4, 18kDa (NADH-coenzyme Q reductase) | -0.72 |
| 8-iso-PGF2a (mmol/mmol creatinine) | *ATP2B4* | ATPase, Ca++ transporting, plasma membrane 4 | -0.73 |
| 8-iso-PGF2a (mmol/mmol creatinine) | *ATP5A1* | ATP synthase, H+ transporting, mitochondrial F1 complex, alpha subunit 1, cardiac muscle | -0.72 |
| 8-iso-PGF2a (mmol/mmol creatinine) | *PAFAH1B1* | platelet-activating factor acetylhydrolase 1b, regulatory subunit 1 (45kDa) | -0.72 |
| 8-iso-PGF2a (mmol/mmol creatinine) | *AK3* | adenylate kinase 3 | -0.74 |
| 8-iso-PGF2a (mmol/mmol creatinine) | *NT5C3* | 5'-nucleotidase, cytosolic III | -0.71 |
| 8-iso-PGF2a (mmol/mmol creatinine) | *ATP5E* | ATP synthase, H+ transporting, mitochondrial F1 complex, epsilon subunit | -0.78 |
| 8-iso-PGF2a (mmol/mmol creatinine) | *ATP5G2* | ATP synthase, H+ transporting, mitochondrial Fo complex, subunit C2 (subunit 9) | -0.73 |
| 8-iso-PGF2a (mmol/mmol creatinine) | *CMPK1* | cytidine monophosphate (UMP-CMP) kinase 1, cytosolic | -0.74 |
| 8-iso-PGF2a (mmol/mmol creatinine) | *ATP5G3* | ATP synthase, H+ transporting, mitochondrial Fo complex, subunit C3 (subunit 9) | -0.71 |
| 8-iso-PGF2a (mmol/mmol creatinine) | *ACP1* | acid phosphatase 1, soluble | -0.71 |
| 8-iso-PGF2a (mmol/mmol creatinine) | *PFKFB4* | 6-phosphofructo-2-kinase/fructose-2,6-biphosphatase 4 | -0.74 |
| 8-iso-PGF2a (mmol/mmol creatinine) | *ATP5J* | ATP synthase, H+ transporting, mitochondrial Fo complex, subunit F6 | -0.75 |
| 8-iso-PGF2a (mmol/mmol creatinine) | *PGK1* | phosphoglycerate kinase 1 | -0.74 |
| 8-iso-PGF2a (mmol/mmol creatinine) | *ATP6V1B2* | ATPase, H+ transporting, lysosomal 56/58kDa, V1 subunit B2 | -0.76 |
| 8-iso-PGF2a (mmol/mmol creatinine) | *PHYH* | phytanoyl-CoA 2-hydroxylase | -0.7 |
| 8-iso-PGF2a (mmol/mmol creatinine) | *PIGH* | phosphatidylinositol glycan anchor biosynthesis, class H | -0.71 |
| 8-iso-PGF2a (mmol/mmol creatinine) | *ATP6V1E1* | ATPase, H+ transporting, lysosomal 31kDa, V1 subunit E1 | -0.8 |
| 8-iso-PGF2a (mmol/mmol creatinine) | *PIK3CA* | phosphoinositide-3-kinase, catalytic, alpha polypeptide | -0.76 |
| 8-iso-PGF2a (mmol/mmol creatinine) | *NUDT9* | nudix (nucleoside diphosphate linked moiety X)-type motif 9 | -0.75 |
| 8-iso-PGF2a (mmol/mmol creatinine) | *ATP5O* | ATP synthase, H+ transporting, mitochondrial F1 complex, O subunit | -0.73 |
| 8-iso-PGF2a (mmol/mmol creatinine) | *A4GALT* | alpha 1,4-galactosyltransferase | 0.74 |
| 8-iso-PGF2a (mmol/mmol creatinine) | *POLB* | polymerase (DNA directed), beta | -0.73 |
| 8-iso-PGF2a (mmol/mmol creatinine) | *MANSC1* | MANSC domain containing 1 | -0.76 |
| 8-iso-PGF2a (mmol/mmol creatinine) | *OXSM* | 3-oxoacyl-ACP synthase, mitochondrial | -0.75 |
| 8-iso-PGF2a (mmol/mmol creatinine) | *MTPAP* | mitochondrial poly(A) polymerase | -0.7 |
| 8-iso-PGF2a (mmol/mmol creatinine) | *PRPS2* | phosphoribosyl pyrophosphate synthetase 2 | -0.75 |
| 8-iso-PGF2a (mmol/mmol creatinine) | *POLE4* | polymerase (DNA-directed), epsilon 4 (p12 subunit) | -0.71 |
| 8-iso-PGF2a (mmol/mmol creatinine) | *CHPT1* | choline phosphotransferase 1 | -0.76 |
| 8-iso-PGF2a (mmol/mmol creatinine) | *PTEN* | phosphatase and tensin homolog | -0.76 |
| 8-iso-PGF2a (mmol/mmol creatinine) | *GALNTL1* | UDP-N-acetyl-alpha-D-galactosamine:polypeptide N-acetylgalactosaminyltransferase-like 1 | 0.75 |
| 8-iso-PGF2a (mmol/mmol creatinine) | *GPAM* | glycerol-3-phosphate acyltransferase, mitochondrial | -0.72 |
| 8-iso-PGF2a (mmol/mmol creatinine) | *SAT1* | spermidine/spermine N1-acetyltransferase 1 | -0.75 |
| 8-iso-PGF2a (mmol/mmol creatinine) | *SDHD* | succinate dehydrogenase complex, subunit D, integral membrane protein | -0.75 |
| 8-iso-PGF2a (mmol/mmol creatinine) | *POLR1E* | polymerase (RNA) I polypeptide E, 53kDa | -0.73 |
| 8-iso-PGF2a (mmol/mmol creatinine) | *BLVRB* | biliverdin reductase B (flavin reductase (NADPH)) | -0.72 |
| 8-iso-PGF2a (mmol/mmol creatinine) | *NADK* | NAD kinase | -0.76 |
| 8-iso-PGF2a (mmol/mmol creatinine) | *CYP4F12* | cytochrome P450, family 4, subfamily F, polypeptide 12 | 0.77 |
| 8-iso-PGF2a (mmol/mmol creatinine) | *SOAT1* | sterol O-acyltransferase 1 | -0.71 |
| 8-iso-PGF2a (mmol/mmol creatinine) | *UAP1* | UDP-N-acteylglucosamine pyrophosphorylase 1 | -0.74 |
| 8-iso-PGF2a (mmol/mmol creatinine) | *TALDO1* | transaldolase 1 | -0.74 |
| 8-iso-PGF2a (mmol/mmol creatinine) | *TPI1* | triosephosphate isomerase 1 | -0.74 |
| 8-iso-PGF2a (mmol/mmol creatinine) | *UGP2* | UDP-glucose pyrophosphorylase 2 | -0.73 |
| 8-iso-PGF2a (mmol/mmol creatinine) | *PPCS* | phosphopantothenoylcysteine synthetase | -0.73 |
| 8-iso-PGF2a (mmol/mmol creatinine) | *UXS1* | UDP-glucuronate decarboxylase 1 | -0.74 |
| 8-iso-PGF2a (mmol/mmol creatinine) | *PLA2G12A* | phospholipase A2, group XIIA | -0.73 |
| 8-iso-PGF2a (mmol/mmol creatinine) | *PIP5K1A* | phosphatidylinositol-4-phosphate 5-kinase, type I, alpha | 0.74 |
| 8-iso-PGF2a (mmol/mmol creatinine) | *POLR3GL* | polymerase (RNA) III (DNA directed) polypeptide G (32kD)-like | -0.71 |
| 8-iso-PGF2a (mmol/mmol creatinine) | *AGPS* | alkylglycerone phosphate synthase | -0.77 |
| 8-iso-PGF2a (mmol/mmol creatinine) | *GFM1* | G elongation factor, mitochondrial 1 | -0.74 |
| 8-iso-PGF2a (mmol/mmol creatinine) | *PLA2G4C* | phospholipase A2, group IVC (cytosolic, calcium-independent) | -0.76 |
| 8-iso-PGF2a (mmol/mmol creatinine) | *AKR1C3* | aldo-keto reductase family 1, member C3 (3-alpha hydroxysteroid dehydrogenase, type II) | -0.72 |
| 8-iso-PGF2a (mmol/mmol creatinine) | *DPM1* | dolichyl-phosphate mannosyltransferase polypeptide 1, catalytic subunit | -0.72 |
| 8-iso-PGF2a (mmol/mmol creatinine) | *PLCZ1* | phospholipase C, zeta 1 | 0.8 |
| 8-iso-PGF2a (mmol/mmol creatinine) | *ATP6V0E1* | ATPase, H+ transporting, lysosomal 9kDa, V0 subunit e1 | -0.77 |
| 8-iso-PGF2a (mmol/mmol creatinine) | *GGPS1* | geranylgeranyl diphosphate synthase 1 | -0.73 |
| 8-iso-PGF2a (mmol/mmol creatinine) | *ATP6V1G1* | ATPase, H+ transporting, lysosomal 13kDa, V1 subunit G1 | -0.74 |
