# Supplementary material for: Transcriptomic Coordination in the Human Metabolic Network Reveals Links between n-3 Fat Intake, Adipose Tissue Gene Expression and Metabolic Health
Source: PLoS Comput Biol. 2011 Nov 3;7(11):e1002223. doi: 10.1371/journal.pcbi.1002223 (PMC3207936; doi:10.1371/journal.pcbi.1002223)
Supplement: Table S6 — Significantly overrepresented Gene Ontology ‘biological process’ terms in the adipose tissue TC network modules. Top 10 terms for each module are shown. (DOCX) [file pcbi.1002223.s008.docx]

**Supplementary Table S6.** Significantly overrepresented Gene Ontology ‘biological process’ terms in the adipose tissue TC network modules. Top 10 terms for each module are shown.

| **Module** | **P value** | **Expected count** | **Observed count** | **Term size** | **Term** |
| --- | --- | --- | --- | --- | --- |
| 1 | 2.23E-11 | 0.4525018 | 9 | 13 | phosphatidylinositol-mediated signaling |
| 1 | 2.91E-11 | 1.5481481 | 14 | 55 | intracellular signal transduction |
| 1 | 1.36E-09 | 0.3132705 | 7 | 9 | phosphatidylinositol phosphorylation |
| 1 | 2.87E-09 | 0.4657534 | 8 | 17 | glycerolipid metabolic process |
| 1 | 6.36E-09 | 0.5221175 | 8 | 15 | phosphatidylinositol biosynthetic process |
| 1 | 1.21E-08 | 1.0094271 | 10 | 29 | second-messenger-mediated signaling |
| 1 | 1.52E-08 | 1.317257 | 11 | 46 | phospholipid metabolic process |
| 1 | 1.82E-08 | 10.1407915 | 27 | 325 | regulation of biological process |
| 1 | 2.23E-08 | 8.2048857 | 24 | 274 | lipid metabolic process |
| 1 | 7.19E-07 | 1.8448151 | 11 | 53 | glycerolipid biosynthetic process |
| 2 | 1.01E-25 | 23.517041 | 74 | 230 | nucleoside phosphate metabolic process |
| 2 | 1.85E-22 | 15.132705 | 56 | 148 | nucleobase, nucleoside, nucleotide and nucleic acid biosynthetic process |
| 2 | 1.08E-20 | 63.802756 | 115 | 624 | cellular nitrogen compound metabolic process |
| 2 | 5.73E-16 | 8.656805 | 36 | 88 | ribonucleotide metabolic process |
| 2 | 6.34E-16 | 8.674672 | 36 | 87 | nucleoside triphosphate metabolic process |
| 2 | 2.59E-14 | 8.588832 | 34 | 84 | purine ribonucleoside triphosphate metabolic process |
| 2 | 8.36E-14 | 6.543872 | 29 | 64 | ribonucleoside triphosphate biosynthetic process |
| 2 | 1.09E-13 | 16.768673 | 48 | 164 | nucleic acid metabolic process |
| 2 | 2.47E-13 | 2.893664 | 19 | 29 | DNA repair |
| 2 | 2.96E-13 | 6.338958 | 28 | 64 | purine ribonucleotide biosynthetic process |
| 3 | 3.77E-17 | 77.018505 | 135 | 377 | cellular ketone metabolic process |
| 3 | 1.24E-11 | 27.432922 | 60 | 130 | organic acid biosynthetic process |
| 3 | 2.87E-10 | 78.107463 | 121 | 392 | oxidation-reduction process |
| 3 | 4.95E-10 | 51.045637 | 88 | 276 | organic acid metabolic process |
| 3 | 2.72E-09 | 14.349529 | 36 | 68 | cellular lipid catabolic process |
| 3 | 9.54E-09 | 8.440899 | 25 | 40 | monocarboxylic acid catabolic process |
| 3 | 3.01E-08 | 8.229877 | 24 | 39 | lipid oxidation |
| 3 | 1.08E-05 | 5.06454 | 15 | 24 | protein homotetramerization |
| 3 | 1.10E-05 | 13.633484 | 29 | 69 | fatty acid metabolic process |
| 3 | 1.89E-05 | 3.165337 | 11 | 15 | branched chain family amino acid catabolic process |
