# Supplementary material for: Transcriptomic Coordination in the Human Metabolic Network Reveals Links between n-3 Fat Intake, Adipose Tissue Gene Expression and Metabolic Health
Source: PLoS Comput Biol. 2011 Nov 3;7(11):e1002223. doi: 10.1371/journal.pcbi.1002223 (PMC3207936; doi:10.1371/journal.pcbi.1002223)
Supplement: Table S7 — Paths detected by applying network analysis algorithm to test muscle tissue dataset (GEO accession GSE474). (DOCX) [file pcbi.1002223.s009.docx]

**Supplementary Table S7.** Paths detected by applying network analysis algorithm to test muscle tissue dataset (GEO accession GSE474).

| **Path #** | **Node1** | **Node2** | **Node3** |
| --- | --- | --- | --- |
| 1 | *NME6* | *EEF1A2* | *RRM1* |
| 2 | *NME6* | *POLE3* | *TRDMT1* |
| 3 | *EEF2* | *NME6* | *PKM2* |
| 4 | *EEF2* | *NME6* | *DNM1L* |
| 5 | *EEF2* | *NME6* | *POLR1E* |
| 6 | *EEF2* | *NME6* | *POLR3K* |
| 7 | *EEF2* | *NME6* | *ADCY2* |
| 8 | *IDH3G* | *GOT1* | *EPRS* |
| 9 | *GAA* | *HK1* | *GFPT1* |
| 10 | *GAA* | *HK1* | *TKT* |
| 11 | *GAA* | *HK1* | *PFKFB3* |
| 12 | *GAA* | *HK1* | *GPI* |
| 13 | *GAA* | *HK1* | *GFPT2* |
| 14 | *ALDOA* | *ALDH6A1* | *ACAA1* |
| 15 | *CERK* | *PPAP2B* | *UGCG* |
| 16 | *CERK* | *PPAP2B* | *SGMS1* |
| 17 | *SAP130* | *HK1* | *GPI* |
| 18 | *CDIPT* | *PIP5K1C* | *PIK3CA* |
| 19 | *CDIPT* | *PIP5K1C* | *PIK3CB* |
| 20 | *ANXA3* | *ADH1B* | *ALDH7A1* |
| 21 | *ANXA3* | *ADH1B* | *ALDH1A3* |
| 22 | *ANXA3* | *ADH1B* | *ALDH3A2* |
| 23 | *ANXA3* | *ADH1B* | *ALDH9A1* |
| 24 | *MTMR12* | *PIK3C3* | *PIKFYVE* |
